# Circulating lymphocyte subsets are prognostic factors in patients with nasopharyngeal carcinoma

**DOI:** 10.1186/s12885-022-09438-y

**Published:** 2022-06-29

**Authors:** Jing Zhu, Ruhua Fang, Zhiwen Pan, Xu Qian

**Affiliations:** Department of Clinical Laboratory, The Cancer Hospital of the University of Chinese Academy of Sciences (Zhejiang Cancer Hospital), Institute of Basic Medicine and Cancer (IBMC), Chinese Academy of Sciences, No 1, East Banshan Road, Gongshu District, Hangzhou, 310022 People’s Republic of China

**Keywords:** Nasopharyngeal carcinoma, Cancer immunity, EBV, Circulating lymphocyte subsets, Chemoradiotherapy, Follow-up

## Abstract

**Background:**

Nasopharyngeal carcinoma (NPC) is a geographically and racially variable disease that has a high incidence in Southeast China. According to previous studies on tumor immunity, we compared multiple clinical parameters and blood indexes with outcomes regarding to Epstein-Barr virus (EBV) status in NPC patients.

**Methods:**

According to the EBV load at diagnosis, 220 NPC patients who received concurrent chemoradiotherapy (CRT) were divided into two groups: EBV DNA ≥ 1500 copies/mL and EBV DNA < 1500 copies/mL, respectively. We compared clinical parameters with peripheral blood mononuclear cells, lymphocyte subsets and biochemical indexes. We also analyzed distant metastases and the overall survival rate regarding to these characteristics.

**Results:**

In most cases, the two groups showed the same trends. Most blood indexes were decreased during CRT and the decrease of the absolute count was more significant than the percentage. Patients with younger age showed the higher CD3+ and CD3 + CD8+ percentages. Patients whose EBV DNA ≥ 1500 copies/mL showed a higher N classification than those with EBV DNA < 1500 copies/mL at first diagnosis. Within patients with EBV DNA ≥ 1500 copies/mL, a higher CD3 + CD8+ percentage or lower CD3-CD56+ percentage had better OS rates, and the CD3 + CD8+ percentage was an independent prognostic factor by multivariate survival analyses.

**Conclusions:**

CRT caused an overall decrease of blood cells in NPC patients. Among all the blood indexes, the CD3 + CD8+ percentage showed a correlation with age and was an independent prognostic factor in patients with EBV DNA ≥ 1500 copies/mL at first diagnosis, which is worthy for further large cohort study.

**Supplementary Information:**

The online version contains supplementary material available at 10.1186/s12885-022-09438-y.

## Background

Nasopharyngeal carcinoma (NPC) is one of malignant epithelial cell tumors at the top and lateral wall of the nasal cavity with different etiology and pathology [1]. According to the World Health Organization (WHO) classification, NPC is divided into keratinizing (K) carcinoma (squamous cell carcinoma; SCC), non-keratinizing (NK) carcinoma (including differentiated and undifferentiated variants) and basaloid squamous cell carcinoma. There are great differences in the geographical and ethnic distributions of NPC. The incidence of NPC is significantly higher in South China and Southeast Asia, mainly belonging to NK-NPC (90%). The population is sensitive to radiotherapy and the disease has almost 100% association with Epstein–Barr virus (EBV) [2]. EBV is a common linear double-stranded virus, about 122–180 nm, which contains a double helix DNA wrapped in the protein capsid. EBV infection has been found mainly in B lymphocytes and epithelial cells which can be cleaved and replicated [3]. The current research on other pathogenic factors of NPC is not thorough. Genetic susceptibility and consumption of pickled food are also important pathogenic factors [2]. Previous studies have demonstrated that high EBV DNA levels may indicate poor prognosis and reduced long-term survival in NPC patients [4, 5]. However, tumor immunity at the circulating level in relation to NPC patient outcome remains to be explored.

Radiotherapy is the main treatment for NPC [6]. Radiotherapy alone is effective in the early stage, concurrent chemoradiotherapy (CRT) in the locally advanced stage, and systematic therapy in patients with metastases and recurrence [7]. In recent years, with the progress of molecular biology, targeted therapy and immunotherapy have become new trends, and have determined their positions as second-line treatments for recurrent/metastatic NPC. Moreover, the exploration of first-line combined chemotherapy also shows a good prospect [8, 9].

Immune cell infiltration is a characteristic of almost all malignant tumors. Immune cells mainly include tumor-associated macrophages, lymphocytes, and mast cells. They play important roles in immune monitoring and contribute to the elimination of tumor cells [10]. Given the minimally invasive nature of blood sample collection compared to tumor infiltrating tissues, the regularity of various indexes in peripheral blood is more convenient for clinical application, such as T lymphocyte cells, B lymphocyte cells and NK cells. The inflammation index is the characteristic marker of tumor and plays an auxiliary role in determining the occurrence and progression of tumor [11]. Peripheral blood Neutrophil count/Lymphocyte count (NLR), Lymphocyte count/Monocyte count (LMR), Platelet count/Lymphocyte count (PLR), Platelet count×Neutrophil count/Lymphocyte count (SII) are widely used indexes in the evaluation of inflammation [12]. In addition, albumin (ALB) and lactate dehydrogenase (LDH) are well-studied prognostic biomarkers of NPC [13].

This study was based on the status of EBV load in patients with NPC. We explored not only the relationship between EBV DNA load, lymphocyte subsets and inflammatory indexes in peripheral blood, but also the relationships between EBV DNA load, clinical parameters, distant metastases and OS. The aim was to investigate indicators with prognostic value in NPC patients, and these findings may hence significantly aid the clinical decision-making process.

## Materials and methods

### Ethics statement and the criteria for patient inclusion

A retrospective single-institution cohort design was used, and a total of 220 patients with NPC who signed an informed consent form and have completed treatment were enrolled in this study. Patients with a history of other malignant tumors were excluded. This study was approved by the local Ethics Committee of the Zhejiang Cancer Hospital (IRB-2021-326). Grading and staging were undertaken according to the 8th edition of the International Union for Cancer Control/American Joint Committee on Cancer (UICC/AJCC) cancer staging system for NPC. Patients who survived were censored at their last date of follow-up. The OS rate was calculated from the date of diagnosis to the date of death.

### Therapy regimen

None of the patients underwent surgery. The tissue sent for pathological diagnosis was obtained by nasopharyngeal biopsy. All patients underwent concurrent CRT. Chemotherapy regimen: Nedaplatin plus Docetaxel; Chemotherapy interval: once every 21 days. Radiotherapy dose: 2.0 Gy. Radiotherapy interval: once every 5 days, suspended for 2 days. Circulating blood samples were collected before CRT. The first time was before therapy, named T1; the second time was during therapy, named T2; the third time was before the last therapy, named T3.

### Blood samples

Circulating peripheral blood mononuclear cells (PBMCs) were obtained during the period between January 2016 and December 2020. Two milliliters of venous blood was collected in a vacuum tube prefilled with EDTA-K_2_ (Zhejiang Gongdong Medical Technology Co., Ltd. China) for EBV DNA detection, white blood cell (WBC) count, neutrophil count, total lymphocyte count and platelet count. Two milliliters of venous blood was collected in a vacuum tube prefilled with heparin sodium (Kangshi Medical Inc., China) for lymphocyte subset detection. Two milliliters of venous blood was collected in a vacuum tube prefilled with coagulant (Zhejiang Gongdong Medical Technology Co., Ltd. China) for ALB and LDH. After centrifugation in Biocoll Separating Solution (Merck, Germany), PBMCs were separated and recovered, washed twice in PBS and stored at 4 °C. Lymphocyte subset detection was performed within 24 h. The patient characteristics are summarized in Table 1.

**Table 1:** Comparison between high-risk group and low-risk group with clinical parameters

**Baseline characteristics**	**High-risk group (*n*=71)** **n (%)**	**Low-risk group (*n*=149)** **n (%)**	***P***
**Age at diagnosis, years**			
<50	31 (43.66)	66 (44.30)	1.000
≥50	40 (56.34)	83 (55.70)	
**Sex**			
Male	53 (74.65)	113 (75.84)	0.868
Female	18 (25.35)	36 (24.16)	
**Smoking**			
Yes	38 (53.52)	74 (49.66)	0.666
No	33 (46.48)	75 (50.34)	
**Drinking**			
Yes	21 (29.58)	42 (28.19)	0.874
No	50 (70.42)	107 (71.81)	
**Family history of cancer**			
Yes	27 (38.03)	53 (35.57)	0.765
No	44 (61.97)	96 (64.43)	
**Pathological type**			
keratinizing	3 (4.23)	17 (11.41)	0.130
non-keratinizing	68 (95.77)	132 (88.59)	
**T classification**			
pT1-2	7 (9.86)	24 (16.11)	0.300
pT3-4	64 (90.14)	125 (83.89)	
**N classification**			
pN0-1	12 (16.90)	38 (25.50)	**<0.001**
pN2-3	59 (83.10)	111 (74.50)	
**UICC stage**			
I-II	3 (4.23)	11 (7.38)	0.556
III-IV	68 (95.77)	138 (92.62)	
**Distant metastases**			
Yes	14 (19.72)	30 (20.13)	1.000
No	57 (80.28)	119 (79.87)	
**Status**			
Dead	12 (16.90)	18 (12.08)	0.401
Alive	59 (83.10)	131 (87.92)	

Data were calculated by Chi-square test.

### EBV DNA and different blood indexes detection

EBV DNA was analyzed by Roche Lightcycler 480 fluorescence quantitative PCR (Roche, USA) with Daan EBV DNA detection reagent (Daan Gene Co., Ltd. China). WBC count, neutrophil count, total lymphocyte count and platelet count were analyzed by a Mindray CAL 8000 automatic blood cell analysis pipeline (Mindray, China) immediately after blood collection. The vacuum tube prefilled with coagulant was centrifuged at 3500 r/min for 5 min, and serum was detected by a Hitachi 7600 automatic biochemical analyzer (HITACHI, Japan). For flow cytometry analysis, PBMCs in 50 μL PBS supplemented with 0.5% BSA (Thermo Fisher, USA) (1 × 10^6^ cells/mL) were incubated with anti-human monoclonal antibodies (mAbs) for 15 min. The following mAbs were used for flow cytometry: anti-CD45/CD4/CD8/CD3, anti-CD45/CD56/CD19/CD3, anti-CD4, anti-CD45RA, anti-CD45RO, anti-CD8 and anti-CD38 (Beckman Coulter, USA) (Supplementary Table 1). OptiLyse C lysing solution (Beckman Coulter, USA) was used for hemolysis for 15 min, and PBMCs were resuspended in PBS (Beckman Coulter, USA). The results were acquired by a Beckman Coulter FC500 flow cytometer and analyzed by CXP analysis software with the recommended reference ranges [12, 14, 15]. The reference ranges were adjusted in our laboratory (Supplementary Table 2).

We divided the patients into two groups: a low-risk group (EBV DNA < 1500 copies/mL at T1) and a high-risk group (EBV DNA ≥ 1500 copies/mL at T1) by a previously defined EBV-DNA cutoff value [16, 17]. We mainly compared the differences between the two groups in the study.

### Statistical analysis

Statistical analyses were performed using STATA 14.2 (Stata Corp LLC, Texas, USA). Categorical variables are described as percentages, and numerical variables are represented as the mean ± SD. Qualitative data were compared using the Chi-square test. Univariate and multivariate analyses were performed by the cox proportional hazards model. Survival was determined using the Kaplan–Meier (KM) method. A *p* value of < 0.05 was regarded as statistically significant.

## Results

### Patient characteristics

A total of 220 patients were included in this study, with a mean age of 52.31 ± 11.10 (ranging from 17 to 79). There were 166 males and 54 females in the study. It takes an average of 3.6 months from the onset of physical discomfort to diagnosis. The clinicopathological parameters of the patients are summarized in Table 1. Most patient tumors were staged as advanced (UICC TNM stages III/IV, 75/131; 93.64%). The median follow-up time was 41.40 ± 16.24 months (range 10–84 months). Some of the patients had a habit of smoking and drinking, and have family history of cancer.

We divided the patients into two groups by EBV DNA load, 149 cases belonged to low-risk group and 71 cases belonged to high-risk group. The two groups showed different in N classification (*p* < 0.001), and the high-risk group showed a higher N classification. The data are shown in Table 1.

Distant metastases were found in 44 patients (20.00%). Among all metastases, 17 were bone, 9 were lung, 6 were liver, 4 were brain, 2 were stomach, 2 were mouth, 1 was uterus and 3 were multiple metastases. All patients with metastatic disease had TNM stage IV disease.

### The relationships between clinical parameters and blood indexes

**Fig. 1:**
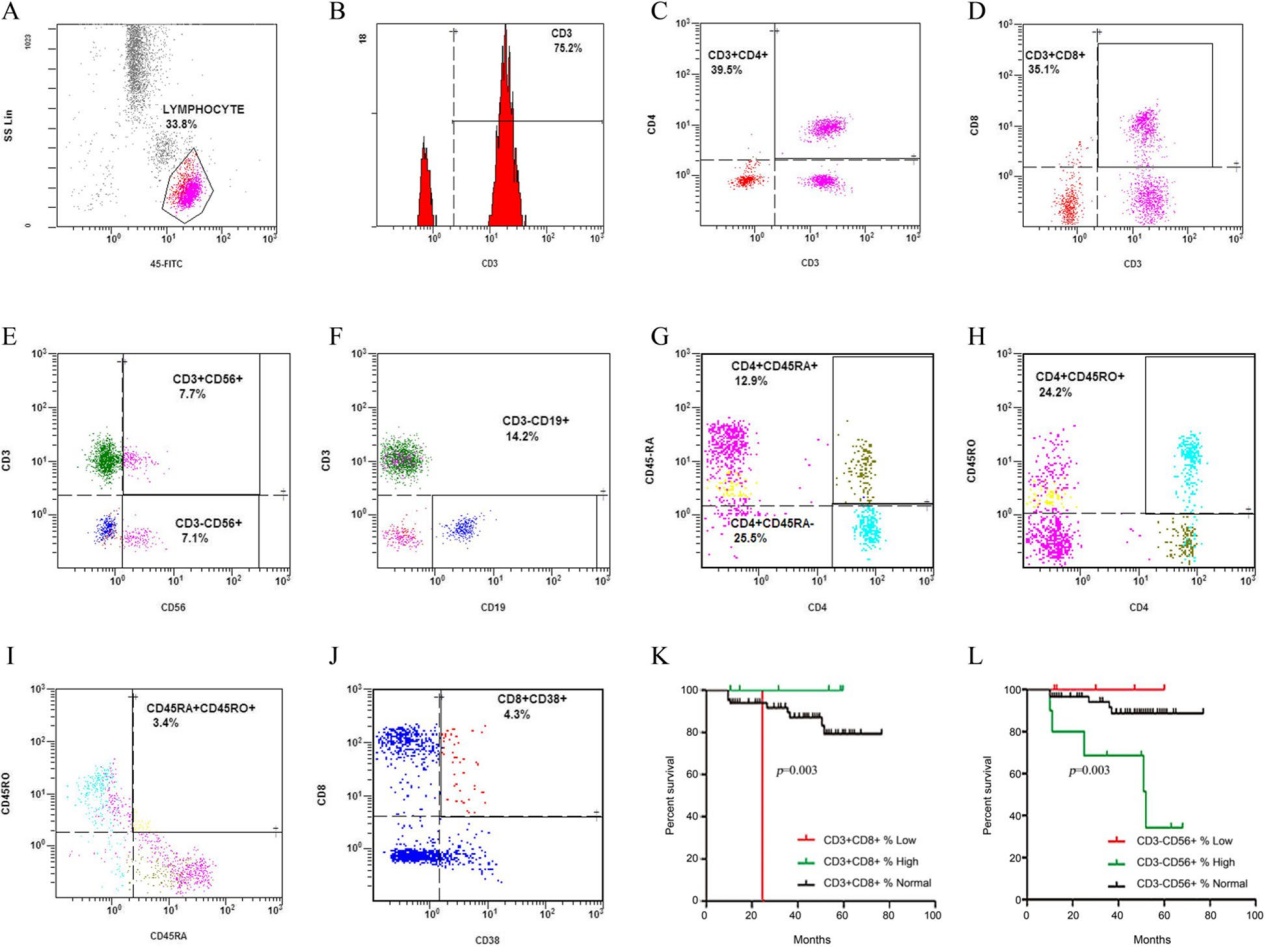
Represented images of flow cytometry results and Kaplan-Meier analysis. A. Lymphocyte percentage; B. CD3+ percentage; C. CD3 + CD4+ percentage; D. CD3 + CD8+ percentage; E. CD3 + CD56+ percentage and CD3-CD56+ percentage; F. CD3-CD19+ percentage; G. CD4 + CD45RA+ percentage and CD4 + CD45RA- percentage; H. CD4 + CD45RO+ percentage; I. CD45RA + CD45RO+ percentage; J. CD8 + CD38+ percentage; K. Kaplan-Meier analysis of CD3 + CD8+ percentage in high-risk group; L: Kaplan-Meier analysis of CD3-CD56+ percentage in high-risk group

We measured EBV DNA load, peripheral blood indexes such as WBC count, neutrophil count, total lymphocyte count, platelet count; lymphocyte subsets such as CD3+ percentage/count, CD3 + CD4+ percentage/count, CD3 + CD8+ percentage/count; biochemical indexes ALB and LDH. The flow cytometry results are shown in Fig. 1A-J.

As shown in Tables 2–3, we studied the relationships between clinical parameters such as age, sex, smoking history, drinking history, family history of cancer, pathological type, clinical stage and the changes of blood indexes in each group. The calculations were carried out at T1.

**Table 2:** Relationship between clinical parameters and lymphocyte subsets in high-risk group before therapy (T1) (*n=*71)

**Parameters**	**Age**	***p***	**Sex**	***p***	**Smoking**	***p***	**Drinking**	***p***	**Family history of cancer**	***p***	**Histology**	***p***	**T classification**	***p***	**N classification**	***p***	**UICC stage**	***p***
	<50 vs ≥50		Male vs Female		Yes vs No		Yes vs No		Yes vs No		K vs NK		pT1-2 vs pT3-4		pN0-1 vs pN2-3		I-II vs III-IV	
CD3+ %	71.05±9.96 67.03±12.34	**.039**	68.89±11.29 67.36±12.81	.632	69.78±11.67 67.10±11.57	.335	65.73±14.93 69.74±9.71	.181	69.30±11.03 67.95±12.11	.635	72.00±3.98 68.29±11.90	.539	71.54±4.65 68.17±12.12	.742	68.34±11.87 68.72±11.47	.234	70.75±6.15 68.43±11.76	.122
CD3+ count	1.22±0.56 1.00±0.33	**.035**	1.08±0.42 1.08±0.49	.950	1.15±0.46 1.01±0.40	.185	1.04±0.43 1.10±0.44	.556	1.08±0.33 1.09±0.50	.946	1.17±0.38 1.08±0.44	.680	1.15±0.60 1.08±0.42	.372	1.10±0.40 1.05±0.49	.624	0.90±0.43 1.09±0.44	.160
CD3+CD4+ %	38.88±7.84 37.92±10.05	.925	38.04±9.41 38.94±9.00	.723	39.68±9.36 36.74±9.02	.183	34.87±9.74 39.80±8.70	.037	38.03±8.63 38.44±9.76	.856	34.83±4.33 38.48±9.44	.447	35.27±8.00 38.60±9.38	.492	39.30±8.23 36.86±10.47	.574	36.50±7.21 38.32±9.34	.507
CD3+CD4+ count	0.65±0.29 0.57±0.22	.366	0.59±0.24 0.62±0.29	.652	0.64±0.26 0.55±0.23	.114	0.55±0.23 0.62±0.26	.234	0.58±0.17 0.61±0.29	.596	0.57±0.20 0.60±0.25	.809	0.59±0.41 0.60±0.23	.207	0.63±0.22 0.56±0.28	.205	0.47±0.27 0.60±0.25	.154
CD3+CD8+ %	26.55±8.40 23.47±9.12	**.014**	25.07±8.96 23.19±8.93	.444	24.49±9.30 24.71±8.63	.918	24.99±9.21 24.42±8.89	.808	25.89±10.22 23.70±7.91	.313	3.30±3.48 24.26±9.05	.190	26.87±5.85 24.35±9.20	.537	23.58±8.93 25.99±8.88	.308	27.00±4.24 24.53±9.03	.814
CD3+CD8+ count	0.48±0.28 0.35±0.19	**.011**	0.41±0.24 0.38±0.24	.660	0.42±0.26 0.38±0.21	.505	0.40±0.24 0.40±0.24	.916	0.41±0.23 0.39±0.24	.663	0.50±0.19 0.39±0.24	.406	0.41±0.19 0.40±0.24	.949	0.39±0.22 0.41±0.26	.710	0.33±0.08 0.40±0.24	.687
CD4/CD8 ratio	1.64±0.76 1.90±0.95	**.034**	1.71±0.73 2.07±1.24	.144	1.84±0.77 1.76±1.01	.741	1.59±0.76 1.90±0.93	.174	1.76±0.96 1.83±0.85	.739	1.18±0.30 1.84±0.90	.148	1.36±0.40 1.85±0.92	.439	1.95±0.96 1.59±0.75	.239	1.35±0.49 1.81±0.90	.726
CD3-CD56+ %	16.42±7.90 20.53±11.40	**.042**	19.28±10.45 18.27±10.48	.724	18.35±9.76 19.75±11.14	.574	21.65±13.34 17.84±8.66	.155	19.46±10.81 18.72±10.21	.773	13.75±5.01 19.34±10.57	.300	16.94±1.71 19.25±10.92	.702	18.34±10.99 19.95±9.62	.232	17.80±1.84 19.06±10.54	.131
CD3-CD56+ count	0.28±0.16 0.33±0.30	.205	0.32±0.27 0.29±0.22	.757	0.30±0.26 0.32±0.26	.836	0.36±0.37 0.29±0.19	.239	0.32±0.27 0.30±0.25	.733	0.24±0.15 0.31±0.26	.580	0.26±0.10 0.32±0.27	.669	0.32±0.28 0.30±0.23	.306	0.22±0.07 0.31±0.26	.394
CD3-CD19+ %	10.58±5.80 9.22±5.00	.505	9.04±3.91 11.71±7.97	.065	9.27±4.86 10.21±5.79	.459	10.19±6.65 9.51±4.64	.622	8.50±3.80 10.56±6.04	.109	10.53±6.39 9.67±5.29	.757	9.37±3.65 9.76±5.48	.988	10.54±5.64 8.59±4.66	.287	9.60±1.98 9.72±5.38	.949
CD3-CD19+ count	0.17±0.09 0.14±0.09	.414	0.14±0.07 0.18±0.13	.124	0.15±0.10 0.14±0.08	.680	0.16±0.12 0.15±0.07	.629	0.13±0.06 0.16±0.11	.093	0.16±0.09 0.15±0.09	.803	0.13±0.03 0.15±0.09	.733	0.17±0.10 0.13±0.08	.083	0.12±0.02 0.15±0.09	.814
CD3+CD56+ %	3.20±2.62 2.56±1.22	.189	2.69±1.32 3.11±2.98	.414	2.65±1.35 2.96±2.32	.497	2.86±1.54 2.77±2.01	.854	2.63±1.33 2.91±2.17	.545	2.13±0.91 2.84±1.91	.463	2.60±1.25 2.82±1.93	.980	3.03±2.17 2.48±1.31	.545	3.30±0.57 2.78±1.89	.399
CD3+CD56+ count	0.06±0.07 0.04±0.02	.196	0.04±0.02 0.06±0.08	.228	0.04±0.03 0.05±0.06	.745	0.05±0.03 0.05±0.05	.959	0.04±0.02 0.05±0.06	.442	0.04±0.02 0.05±0.05	.681	0.04±0.01 0.05±0.05	.819	0.05±0.06 0.04±0.02	.260	0.04±0.01 0.05±0.05	.289
CD4+CD45RA+ %	12.79±4.03 8.45±5.02	**.026**	9.58±5.23 11.40±4.61	.194	10.16±5.40 9.91±4.85	.836	8.40±5.60 10.78±4.75	.070	10.06±4.37 10.03±5.62	.981	10.25±2.79 10.03±5.23	.934	11.31±4.94 9.90±5.15	.700	10.88±4.51 8.90±5.72	.390	12.55±3.18 9.97±5.15	**.049**
CD4+CD45RA+ count	0.22±0.13 0.13±0.10	**.040**	0.16±0.12 0.19±0.11	.349	0.18±0.13 0.15±0.10	.354	0.14±0.12 0.17±0.12	.229	0.15±0.08 0.17±0.14	.642	0.17±0.07 0.16±0.12	.924	0.20±0.19 0.16±0.11	.212	0.18±0.10 0.14±0.14	.321	0.16±0.10 0.16±0.12	**.047**
CD4+CD45RA- %	22.60±5.99 24.94±7.73	.220	24.20±7.35 23.74±6.86	.818	25.32±7.26 22.74±6.96	.131	22.85±7.24 24.64±7.17	.334	23.82±6.36 24.26±7.78	.802	20.78±5.69 24.28±7.25	.347	20.33±5.80 24.50±7.24	.350	24.59±6.04 23.39±8.57	.141	21.95±5.30 24.15±7.25	.853
CD4+CD45RA- count	0.38±0.17 0.37±0.14	.937	0.37±0.14 0.38±0.17	.935	0.40±0.15 0.34±0.15	.085	0.36±0.15 0.38±0.15	.496	0.36±0.11 0.38±0.17	.625	0.34±0.14 0.38±0.15	.606	0.34±0.22 0.38±0.14	.239	0.39±0.13 0.35±0.17	.370	0.29±0.17 0.38±0.15	.364
CD4+CD45RA+/CD4+CD45RA- ratio	0.60±0.24 0.36±0.22	**.003**	0.42±0.25 0.52±0.25	.194	0.43±0.26 0.47±0.25	.470	0.39±0.28 0.47±0.24	.192	0.46±0.27 0.44±0.24	.644	0.55±0.30 0.44±0.25	.426	0.57±0.21 0.43±0.26	.546	0.47±0.22 0.42±0.29	.609	0.57±0.01 0.44±0.26	.233
CD4+CD45RO+ %	22.43±6.03 24.79±7.68	.244	23.93±7.32 23.91±6.90	.801	25.01±7.27 22.74±6.97	.112	22.82±7.23 24.42±7.16	.322	23.64±6.51 24.12±7.66	.831	20.48±0.24 24.13±7.25	.320	20.14±5.21 24.34±7.26	.331	24.54±6.05 23.09±8.51	.123	21.40±3.39 24.00±7.25	.821
CD4+CD45RO+ count	0.37±0.16 0.37±0.14	.902	0.37±0.14 0.38±0.17	.930	0.40±0.14 0.34±0.15	.092	0..36±0.15 0.38±0.15	.471	0.36±0.11 0.38±0.17	.611	0.33±0.13 0.37±0.15	.611	0.34±0.21 0.37±0.14	.208	0.39±0.13 0.35±0.17	.314	0.28±0.15 0.37±0.15	.355
CD8+CD38+ %	5.78±2.61 6.33±3.27	.654	6.34±3.13 5.51±2.75	.319	5.86±2.96 6.41±3.14	.447	6.57±3.24 5.93±2.96	.415	6.38±3.26 5.95±2.90	.558	5.18±1.84 6.18±3.09	.523	6.39±2.49 6.10±3.11	.317	5.92±3.18 6.40±2.86	.230	7.30±0.42 6.09±3.07	.149
CD8+CD38+ count	0.10±0.06 0.10±0.08	.996	0.10±0.08 0.09±0.07	.489	0.10±0.07 0.10±0.08	.765	0.11±0.09 0.09±0.06	.407	0.10±0.07 0.10±0.08	.819	0.09±0.06 0.10±0.08	.809	0.09±0.05 0.10±0.08	.511	0.10±0.08 0.10±0.07	.181	0.09±0.04 0.10±0.08	.459
WBC count	6.83±1.80 6.58±2.14	1.000	6.80±2.13 6.31±1.64	.380	6.62±1.93 6.74±2.13	.809	6.77±2.28 6.63±1.91	.787	6.50±2.07 6.80±1.99	.539	6.23±0.61 6.70±2.07	.649	7.00±3.54 6.64±1.82	.413	6.10±1.60 7.46±2.27	**.016**	5.90±0.85 6.70±2.04	.353
Neutrophil count	4.51±1.45 4.57±2.18	.557	4.65±2.08 4.23±1.38	.428	4.42±1.81 4.23±1.38	.561	4.65±2.21 4.50±1.81	.758	4.40±2.06 4.65±1.85	.595	3.95±0.58 4.58±1.98	.528	4.77±3.51 3.02±1.46	.682	3.25±1.55 2.73±1.30	**.021**	2.23±0.61 3.05±1.47	.262
NLR	3.13±1.66 3.63±2.97	.273	3.52±2.78 3.22±1.84	.665	3.13±2.09 3.78±3.00	.291	3.74±3.40 3.31±2.13	.517	3.09±2.24 3.69±2.77	.334	2.62±1.32 3.49±2.62	.511	4.43±5.95 4.52±1.72	.588	3.97±1.34 5.34±2.32	.096	4.10±0.28 4.56±1.96	.227
Monocyte count	0.63±0.20 0.52±0.22	**.022**	0.57±0.23 0.51±0.19	.312	0.56±0.22 0.55±0.21	.823	0.59±0.24 0.54±0.21	.381	0.49±0.20 0.60±0.22	**.039**	0.55±0.17 0.56±0.22	.955	0.60±0.28 0.03±0.18	.626	0.02±0.16 0.03±0.18	.088	0.00±0.00 0.03±0.17	.892
LMR	2.77±0.97 3.18±1.67	.148	2.95±1.50 3.26±1.35	.449	3.03±1.58 3.03±1.35	.987	2.97±1.78 3.05±1.31	.826	3.42±1.76 2.76±1.16	.058	3.16±0.85 3.02±1.49	.852	3.14±1.55 3.34±1.96	.901	2.82±1.48 4.30±3.41	.316	3.51±1.16 3.44±2.60	.156
Platelet count	271.00±78.31 218.62±61.59	**.017**	225.45±65.3 274.17±81.1	**.012**	224.03±73.92 252.79±68.33	.094	225.09±75.31 243.51±70.89	.324	234.14±75.51 240.33±70.73	.725	217.50±91.30 239.01±71.63	.567	208.29±73.94 229.28±74.20	.248	210.34±50.11 263.23±117.77	.281	205.00±25.46 233.49±89.85	.195
PLR	189.98±102.5 161.59±72.8	.344	159.97±69.4 207.35±115.9	**.041**	152.83±68.16 192.83±97.47	.048	168.23±81.97 173.67±87.51	.805	158.44±68.14 181.34±95.01	.269	137.92±69.29 174.02±86.15	.415	157.01±105.76 173.62±83.60	.265	166.70±86.60 179.21±84.37	.775	147.12±62.22 172.71±86.09	.152
SII	873.96±569.6 820.65±815.0	.863	812.6±760.9 921.4±647.4	.589	713.74±575.1 977.75±857.2	.129	868.2±929.6 827.6±632.7	.831	739.94±630.8 909.38±792.6	.341	566.95±321.5 856.48±746.3	.446	1034.0±1632.8 818.97±578.7	.498	684.16±480.17 1053.38±943.3	.200	594.39±213.5 847.29±739.8	.146
ALB	42.05±4.04 42.70±3.84	.600	42.92±3.82 41.09±3.92	.085	43.49±3.79 41.34±3.76	**.019**	43.04±3.16 42.20±4.20	.404	42.33±4.01 42.55±3.87	.815	41.25±5.84 42.53±3.81	.527	41.06±3.60 42.61±3.93	.230	42.83±3.34 41.95±4.57	.522	42.90±2.83 42.45±3.94	.719
LDH	219.92±53.2 240.07±103.7	.205	230.92±94.2 237.89±72.4	.776	232.32±78.04 233.09±100.3	.971	251.6±105.1 224.2±80.1	.232	228.62±86.35 235.50±91.28	.751	214.50±44.03 233.78±90.78	.676	263.86±181.1 241.03±71.9	.375	233.98±70.78 243.03±75.12	.095	168.50±4.95 239.81±72.3	.101

Abbreviations: *K* keratinizing, *NK* non-keratinizing, *NLR* neutrophil count/Lymphocyte count, *LMR* lymphocyte count/monocyte count, *PLR* Platelet count/Lymphocyte count, *SII* Platelet count×Neutrophil count/Lymphocyte count, *ALB* albumin, *LDH* lactate dehydrogenase

**Table 3:** Relationship between clinical parameters and lymphocyte subsets in low-risk group before therapy (T1) (*n=*149)

**Parameters**	**Age**	***p***	**Sex**	***p***	**Smoking**	***p***	**Drinking**	***p***	**Family history of cancer**	***p***	**Histology**	***p***	**T classification**	***p***	**N classification**	***p***	**UICC stage**	***p***
	<50 vs ≥50		Male vs Female		Yes vs No		Yes vs No		Yes vs No		K vs NK		pT1-2 vs pT3-4		pN0-1 vs pN2-3		I-II vs III-IV	
CD3+ %	72.35±9.17 67.26±10.74	**.011**	68.94±10.35 70.84±10.50	.336	68.13±10.80 70.69±9.87	.133	68.07±9.95 69.94±10.55	.324	68.49±9.11 69.92±11.04	.423	67.18±9.76 69.70±10.46	.347	68.70±9.88 69.54±10.51	.726	68.75±11.67 69.64±9.95	.744	68.20±8.81 69.51±10.52	.301
CD3+ count	1.19±0.40 1.15±0.41	.666	1.20±0.41 1.07±0.39	.101	1.22±0.41 1.11±0.40	.107	1.17±0.41 1.16±0.41	.894	1.21±0.41 1.14±0.40	.310	1.22±0.40 1.16±0.41	.569	1.22±0.37 1.16±0.42	.910	1.05±0.43 1.21±0.39	.216	1.15±0.44 1.17±0.41	.406
CD3+CD4+ %	38.26±8.58 38.26±8.49	.647	38.47±8.85 37.61±7.45	.594	38.73±8.31 37.80±8.72	.508	38.84±7.60 38.03±8.86	.605	38.37±9.08 38.20±8.22	.908	37.58±7.66 38.35±8.63	.726	38.77±7.99 38.17±8.62	.990	38.35±9.11 38.23±8.33	.886	39.61±8.67 38.15±8.51	.941
CD3+CD4+ count	0.63±0.25 0.65±0.25	.466	0.67±0.26 0.57±0.21	**.029**	0.69±0.25 0.60±0.25	**.020**	0.68±0.27 0.63±0.24	.347	0.68±0.27 0.62±0.24	.160	0.69±0.25 0.64±0.25	.466	0.69±0.24 0.64±0.25	.799	0.59±0.26 0.66±0.25	.423	0.68±0.31 0.64±0.25	.656
CD3+CD8+ %	28.22±8.19 24.79±7.58	**.011**	25.55±7.69 28.32±8.65	.068	24.99±7.89 27.48±7.97	.057	25.30±7.44 26.61±8.21	.370	25.20±8.42 26.82±7.74	.240	24.58±6.39 26.46±8.18	.364	25.84±7.66 26.31±8.09	.526	25.13±7.51 26.62±8.16	.606	23.35±6.09 26.47±8.10	.158
CD3+CD8+ count	0.46±0.20 0.43±0.20	.255	0.44±0.20 0.43±0.20	.759	0.45±0.21 0.43±0.19	.539	0.43±0.17 0.45±0.21	.662	0.44±0.21 0.44±0.20	.916	0.45±0.19 0.44±0.20	.847	0.46±0.19 0.44±0.21	.822	0.38±0.19 0.46±0.20	.168	0.39±0.15 0.45±0.21	.277
CD4/CD8 ratio	1.52±0.71 1.72±0.74	.055	1.68±0.76 1.49±0.63	.157	1.73±0.77 1.54±0.68	.099	1.68±0.62 1.62±0.77	.644	1.77±0.91 1.56±0.61	.097	1.65±0.57 1.63±0.75	.939	1.68±0.79 1.63±0.72	.734	1.68±0.65 1.62±0.76	.845	1.84±0.66 1.62±0.74	.149
CD3-CD56+ %	15.49±8.13 19.7±9.93	**.027**	18.37±9.56 16.57±8.96	.315	19.28±9.55 16.58±9.14	.080	19.40±9.44 17.34±9.38	.230	18.79±8.42 17.44±9.93	.407	20.22±10.83 17.63±9.22	.286	19.52±10.57 17.63±9.20	.605	18.92±10.95 17.58±8.86	.228	20.74±11.07 17.7±9.28	.110
CD3-CD56+ count	0.26±0.20 0.33±0.20	.096	0.32±0.21 0.25±0.16	.055	0.35±0.21 0.26±0.18	**.010**	0.33±0.21 0.29±0.20	.250	0.34±0.21 0.28±0.19	.090	0.39±0.27 0.29±0.19	.054	0.36±0.24 0.29±0.19	.502	0.28±0.18 0.31±0.21	.466	0.35±0.25 0.30±0.20	.792
CD3-CD19+ %	9.17±4.11 9.93±4.31	.215	9.80±4.37 9.03±3.77	.341	9.49±4.68 9.73±3.76	.724	9.95±4.75 9.48±4.02	.542	9.62±3.87 9.6±4.43	.983	10.07±4.20 9.55±4.24	.634	9.60±4.12 9.61±4.26	.138	9.43±4.38 9.67±4.19	.283	9.19±4.03 9.64±4.25	.059
CD3-CD19+ count	0.15±0.09 0.17±0.10	.158	0.17±0.10 0.13±0.08	**.034**	0.17±0.11 0.15±0.08	.198	0.17±0.11 0.16±0.09	.437	0.17±0.09 0.16±0.10	.475	0.19±0.12 0.16±0.09	.144	0.17±0.09 0.16±0.10	.406	0.14±0.09 0.17±0.10	.159	0.15±0.08 0.16±0.10	.146
CD3+CD56+ %	2.94±1.58 2.66±2.18	.528	2.81±2.09 2.67±1.47	.693	2.79±2.29 2.77±1.56	.939	2.94±2.77 2.72±1.52	.538	2.49±1.35 2.93±2.20	.188	2.95±1.49 2.76±2.00	.695	2.46±1.27 2.84±2.05	.132	2.73±1.40 2.79±2.11	.921	2.61±1.17 2.79±2.00	.247
CD3+CD56+ count	0.05±0.03 0.05±0.04	.837	0.05±0.04 0.04±0.03	.209	0.05±0.04 0.04±0.03	.184	0.05±0.05 0.04±0.03	.296	0.04±0.02 0.05±0.04	.352	0.06±0.03 0.05±0.04	.234	0.04±0.03 0.05±0.04	.280	0.04±0.02 0.05±0.04	.716	0.04±0.03 0.05±0.04	.230
CD4+CD45RA+ %	11.85±6.06 10.79±5.67	.706	11.31±5.99 11.02±5.45	.793	11.26±5.85 11.22±5.87	.967	11.15±5.38 11.28±6.04	.908	11.84±6.07 10.91±5.72	.356	11.92±5.59 11.15±5.89	.614	10.63±4.90 11.35±6.01	.951	11.57±6.60 11.13±5.59	.815	10.11±4.68 11.33±5.93	.723
CD4+CD45RA+ count	0.19±0.11 0.18±0.12	.898	0.20±0.12 0.16±0.10	.156	0.20±0.12 0.18±0.11	.229	0.20±0.12 0.18±0.11	.590	0.21±0.13 0.18±0.11	.091	0.20±0.07 0.19±0.12	.603	0.19±0.12 0.19±0.12	.976	0.18±0.12 0.19±0.11	.874	0.17±0.11 0.19±0.12	.742
CD4+CD45RA- %	21.37±6.04 22.36±5.00	.276	21.78±5.62 22.42±5.02	.537	22.11±4.98 21.78±5.93	.712	22.60±4.17 21.68±5.90	.358	21.49±5.43 22.19±5.50	.459	20.66±5.22 22.11±5.49	.308	22.71±5.79 21.80±5.42	.598	20.49±4.65 22.44±5.65	.100	24.25±6.11 21.76±5.39	.135
CD4+CD45RA- count	0.35±0.17 0.38±0.15	.326	0.38±0.16 0.34±0.13	.144	0.40±0.16 0.34±0.14	**.019**	0.40±0.17 0.36±0.15	.227	0.38±0.16 0.36±0.15	.476	0.39±0.19 0.37±0.15	.613	0.40±0.14 0.37±0.16	.646	0.31±0.14 0.39±0.16	**.025**	0.41±0.19 0.37±0.15	.476
CD4+CD45RA+/CD4+CD45RA- ratio	0.60±0.39 0.50±0.29	.303	0.55±0.36 0.52±0.28	.561	0.54±0.37 0.55±0.31	.957	0.50±0.24 0.56±0.37	.301	0.59±0.41 0.52±0.29	.186	0.62±0.36 0.53±0.34	.302	0.50±0.26 0.55±0.35	.769	0.62±0.46 0.52±0.28	.242	0.42±0.19 0.55±0.35	.309
CD4+CD45RO+ %	21.17±6.26 22.28±5.19	.209	21.69±5.84 22.16±5.19	.511	22.02±5.20 21.60±6.13	.689	22.41±4.34 21.57±6.12	.329	21.17±5.57 20.46±5.42	.422	20.46±5.42 21.98±5.70	.321	22.63±5.98 21.66±5.62	.567	20.42±4.82 22.28±5.88	.176	24.15±6.48 21.62±5.58	.130
CD4+CD45RO+ count	0.35±0.17 0.38±0.15	.301	0.38±0.17 0.33±0.13	.129	0.40±0.17 0.34±0.14	**.017**	0.39±0.17 0.36±0.16	.205	0.38±0.16 0.36±0.16	.445	0.39±0.19 0.37±0.16	.605	0.40±0.15 0.36±0.16	.633	0.31±0.14 0.39±0.16	**.036**	0.41±0.20 0.37±0.16	.452
CD8+CD38+ %	5.80±2.81 6.70±4.22	.149	6.42±4.05 6.01±2.42	.556	6.55±4.47 6.09±2.78	.451	5.90±3.26 6.49±3.87	.385	6.50±3.02 6.22±4.05	.666	7.50±3.94 6.17±3.67	.164	6.29±2.83 6.32±3.86	1.000	6.51±4.92 6.25±3.22	.846	6.19±2.32 6.33±3.80	.934
CD8+CD38+ count	0.10±0.07 0.11±0.08	.273	0.11±0.08 0.09±0.04	.096	0.12±0.09 0.09±0.06	**.045**	0.11±0.08 0.11±0.07	.946	0.11±0.07 0.10±0.08	.374	0.14±0.09 0.10±0.07	.090	0.11±0.07 0.11±0.08	.952	0.10±0.08 0.11±0.07	.843	0.10±0.05 0.11±0.08	.794
WBC count	6.66±1.80 6.51±2.02	**.948**	6.88±1.92 5.67±1.66	**<.001**	6.86±1.94 6.29±1.88	.068	6.50±2.06 6.60±1.88	.781	6.62±1.58 6.55±2.10	.845	6.91±1.96 6.53±1.93	.446	6.27±1.77 6.63±1.96	.812	6.01±1.60 6.77±2.00	.169	5.77±1.64 6.64±1.94	.282
Neutrophil count	4.42±1.44 4.22±1.68	.790	4.50±1.61 3.70±1.34	**.007**	4.42±1.60 4.19±1.56	.366	4.18±1.61 4.35±1.57	.563	4.19±1.23 4.37±1.75	.512	4.33±1.64 4.30±1.58	.943	3.86±1.30 4.38±1.62	.432	3.97±1.37 4.42±1.64	.422	3.46±1.01 4.37±1.60	.152
NLR	2.90±1.16 2.64±1.17	.399	2.77±1.15 2.68±1.25	.695	2.62±1.07 2.88±1.25	.173	2.55±0.91 2.83±1.25	.202	2.59±1.14 2.83±1.18	.230	2.61±1.30 2.77±1.16	.610	2.25±0.72 2.84±1.21	.145	2.80±1.03 2.73±1.22	.881	2.19±0.63 2.79±1.19	.261
Monocyte count	0.61±0.31 0.59±0.31	.600	0.64±0.32 0.46±0.23	**.002**	0.67±0.30 0.53±0.30	**.003**	0.60±0.27 0.60±0.32	.881	0.65±0.34 0.57±0.29	.100	0.74±0.48 0.58±0.28	.053	0.63±0.27 0.59±0.32	.947	0.52±0.14 0.63±0.35	.297	0.63±0.26 0.60±0.31	.966
LMR	3.06±1.58 3.15±1.39	.645	2.83±1.13 3.96±1.98	**<.001**	2.78±1.10 3.44±1.70	**.005**	3.01±1.28 3.15±1.54	.583	3.09±1.20 3.13±1.60	.870	3.02±1.11 3.12±1.51	.785	3.22±1.36 3.09±1.49	.710	3.09±1.11 3.12±1.57	.111	2.81±0.89 3.14±1.50	.201
Platelet count	243.29±73.56 224.67±61.31	.067	237.46±66.58 217.68±67.61	.121	234.78±63.22 230.33±71.19	.687	238.14±71.64 230.35±65.54	.526	235.74±62.18 230.78±70.01	.668	219.00±63.99 234.29±67.59	.379	234.30±64.34 232.22±67.90	.107	224.18±58.13 235.41±69.99	.731	227.00±65.20 232.99±67.52	.181
PLR	162.06±65.31 141.9±54.91	.074	147.51±58.01 159.24±66.30	.338	141.73±56.83 158.99±62.46	.593	151.10±67.92 150.15±57.17	.499	145.11±53.48 153.35±63.62	.279	129.38±46.85 153.13±61.28	.328	139.81±47.86 152.36±62.11	.228	160.71±57.39 146.90±60.92	.465	146.68±55.69 150.72±60.68	.125
SII	707.31±336.55 592.26±302.43	**.033**	655.44±313.2 596.91±345.40	.306	626.69±319.88 654.94±324.23	.080	612.37±298.22 652.11±330.59	.932	602.41±285.09 662.16±339.19	.425	568.91±307.30 650.18±323.01	.126	543.90±267.39 658.61±328.02	.092	628.82±267.55 645.04±338.75	.906	498.00±196.81 652.30±326.99	.317
ALB	44.68±5.52 43.06±5.17	.085	43.91±5.88 43.23±3.38	.504	43.60±5.04 43.89±5.70	.741	43.59±3.12 43.80±6.03	.831	43.16±3.67 44.06±6.10	.327	41.51±4.92 44.03±5.37	.068	44.24±3.07 43.65±5.69	.960	45.33±8.15 43.20±3.90	.090	43.83±1.98 43.74±5.55	.974
LDH	196.13±49.84 204.29±49.44	.248	199.19±44.13 205.84±63.84	.481	198.43±38.58 203.21±58.66	.558	199.93±39.08 201.20±53.33	.889	202.04±48.18 200.18±50.61	.827	208.24±73.29 199.89±46	.516	185.35±31.72 203.67±51.81	.248	188.37±36.37 205.11±52.85	.270	184.64±34.78 202.13±50.47	.620

Abbreviations: *K* keratinizing, *NK* non-keratinizing, *NLR* neutrophil count/Lymphocyte count, *LMR* lymphocyte count/monocyte count, *PLR* Platelet count/Lymphocyte count, *SII* Platelet count×Neutrophil count/Lymphocyte count, *ALB* albumin, *LDH* lactate dehydrogenase

Among the clinical parameters, age was more correlated with lymphocyte subsets. The significant differences throughout CRT were CD3+ percentage (the younger age, the higher CD3+ percentage), and CD3 + CD8+ percentage (the younger age, the higher CD3 + CD8+ percentage). The other clinical parameters and blood indexes didn’t always show the same rule throughout the therapy. For example, in the high-risk group, patients with higher N classification showed higher WBC count and neutrophil count. In low-risk group, patients with higher N classification showed higher CD4 + CD45RA- count and CD4 + CD45RO+ count.

### The relationships between EBV DNA and blood indexes

The relationships between EBV DNA load and lymphocyte subsets at T1 were as follows: a higher EBV DNA load was correlated with higher CD4 + CD45RA- percentage (*p* = 0.015), CD4 + CD45RO+ percentage (*p* = 0.019) and lower CD4 + CD45RA+/CD4 + CD45RA- ratio (*p* = 0.034) (Supplementary Table 3). Additionally, patients with a higher EBV load (high-risk group) had higher NLR (*p* = 0.009), PLR (*p* = 0.021) and SII (*p* = 0.004) compared to the low-risk group. Furthermore, the high-risk group demonstrated higher plasma LDH levels compared to the low-risk group (*p* < 0.001) (Supplementary Table 3). At T2 and T3, these correlations were not observed. Data were presented as the mean value and were shown in Supplementary Tables 3, 4, 5 and 6.

### Expression of blood indexes over time

When we compared the high-risk group and the low-risk group, we found that the two groups were consistent during CRT. The decrease in absolute count was more significant than that in percentage, and all lymphocyte subset counts were decreased at the end of therapy. Additionally, the CD4/CD8 ratio and the CD4 + CD45RA+/CD4 + CD45RA- ratios were significantly decreased during therapy. But NLR (*p* < 0.001), PLR (*p* < 0.001) and SII (*p* < 0.001) showed different patterns with that ratios increased over time. These changes were more significant in the low-risk group. Data were shown in Supplementary Tables 3, 4, 5 and 6.

### Expression of blood indexes in patients with or without distant metastases

In 44 patients with distant metastases, 14 belonged to the high-risk group and 30 belonged to the low-risk group. In the high-risk group, monocyte count showed lower in patients with distant metastases at T1. In low-risk group, the expression of CD3+ percentage and PLR were higher while CD3-CD56+ count and CD8 + CD38+ count were lower in patients with distant metastases at T1. The data were shown in Supplementary Tables 7 and 8.

### Overall survival

In the high-risk group, after a median follow-up of 39.24 months, the OS rate was 83.10%. In the low-risk group, after a median follow-up of 42.44 months, the OS rate was 87.92%. EBV DNA load was not an independent prognostic factor for NPC in our analysis (data not shown).

We explored the role of clinical parameters and blood indexes in predicting survival before therapy. In the high-risk group, in univariate survival analyses, we found that age (HR = 1.059, 95% CI: 1.000–1.120, *p* = 0.049), CD3+ percentage (HR = 0.951, 95% CI: 0.915–0.988, *p* = 0.010), CD3 + CD8+ percentage (HR = 0.902, 95% CI: 0.818–0.995, *p* = 0.039) and CD3-CD56+ percentage (HR = 1.045, 95% CI: 1.010–1.081, *p* = 0.011) were significant factors related to survival. In multivariate survival analyses, CD3 + CD8+ percentage (HR = 0.917, 95% CI: 0.810–1.038, *p* = 0.034) was the only factor associated with survival. The data were shown in Table 4. In the low-risk group, in univariate survival analyses, we found that age (HR = 1.078, 95% CI: 1.022–1.136, *p* = 0.005), CD4/CD8 ratio (HR = 1.834, 95% CI: 1.054–3.192, *p* = 0.032), CD4 + CD45RA- % (HR = 1.083, 95% CI: 1.010–1.160, *p* = 0.025), CD4 + CD45RO+ % (HR = 1.090, 95% CI: 1.015–1.171, *p* = 0.018) and Platelet count (HR = 1.003, 95% CI: 1.000–1.007, *p* = 0.042) were significant factors related to survival. In multivariate survival analyses, age (HR = 1.089, 95% CI: 1.026–1.156, *p* = 0.005) and Platelet count (HR = 1.004, 95% CI: 1.000–1.008, *p* = 0.030) were significant factors associated with survival. The data were shown in Supplementary Table 9. The EBV status may contribute to the differences of independent factors between the two groups. In addition, our analysis did not show the significance of TNM staging to predict survival. One reason is that the study population consisted of mainly stage III and IV patients in our study. A future study including more stage I and II patients is warranted.

**Table 4:** Univariate and multivariate survival analyses of overall survival in high-risk group (*n=*71)

**Variable**	**Univariate**		**Multivariate**	
	***p***	**HR (95% CI)**	***p***	**HR (95% CI)**
Age	**0.049**	1.059 (1.000-1.120)	0.063	1.032 (0.970-1.098)
Sex	0.760	1.236 (0.318-4.802)		
Smoking	0.476	1.585 (0.446-5.626)		
Drinking	0.791	0.832 (0.214-3.229)		
Family history of cancer	0.611	0.703 (0.181-2.730)		
T classification	0.924	1.301 (1.002-1.425)		
N classification	0.944	1.560 (0.700-1.998)		
UICC stage	0.179	1.896 (0.536-2.313)		
Distant metastases	0.076	1.145 (0.886-1.160)		
Lymphocyte count	0.612	0.683 (0.156-2.988)		
CD3+ %	**0.010**	0.951 (0.915-0.988)	0.097	0.965 (0.872-1.068)
CD3+ count	0.140	0.282 (0.052-1.515)		
CD3+CD4+ %	0.331	0.974 (0.924-1.027)		
CD3+CD4+ count	0.216	0.170 (0.010-2.803)		
CD3+CD8+ %	**0.039**	0.902 (0.818-0.995)	**0.034**	0.917 (0.810-1.038)
CD3+CD8+ count	0.143	0.048 (0.001-2.805)		
CD4/CD8 ratio	0.353	1.467 (0.654-3.291)		
CD3-CD56+ %	**0.011**	1.045 (1.010-1.081)	0.195	0.999 (0.919-1.086)
CD3-CD56+ count	0.115	2.196 (0.583-4.954)		
CD3-CD19+ %	0.993	0.999 (0.907-1.101)		
CD3-CD19+ count	0.458	0.007 (0.009-1.510)		
CD3+CD56+ %	0.096	0.653 (0.395-1.079)		
CD3+CD56+ count	0.185	0.702 (0.053-1.256)		
CD4+CD45RA+ %	0.173	0.910 (0.794-1.042)		
CD4+CD45RA+ count	0.131	0.430 (0.260-1.692)		
CD4+CD45RA- %	0.564	0.978 (0.905-1.056)		
CD4+CD45RA- count	0.251	0.668 (0.071-3.681)		
CD4+CD45RA+/CD4+CD45RA- ratio	0.218	0.149 (0.007-3.079)		
CD4+CD45RO+ %	0.328	0.960 (0.885-1.042)		
CD4+CD45RO+ count	0.251	0.068 (0.001-6.681)		
CD8+CD38+ %	0.748	1.021 (0.901-1.157)		
CD8+CD38+ count	0.804	0.309 (0.043-2.341)		
WBC count	0.836	1.037 (0.733-1.468)		
Neutrophil count	0.795	1.051 (0.725-1.523)		
NLR	0.584	1.047 (0.889-1.232)		
Monocyte count	0.513	2.454 (0.167-3.111)		
LMR	0.173	0.718 (0.446-1.156)		
Platelet count	0.432	1.002 (0.997-1.006)		
PLR	0.439	1.001 (0.999-1.003)		
SII	0.276	1.000 (1.000-1.001)		
LDH	0.887	0.998 (0.971-1.026)		
LDH	0.497	0.994 (0.978-1.011)		

*Abbreviations*: *HR* hazard ratio, *CI* confidence interval, *NLR* Neutrophil count/Lymphocyte count, *LMR* Lymphocyte count/Monocyte count, *PLR* Platelet count/Lymphocyte count, *SII* Platelet count×Neutrophil count/Lymphocyte count, *ALB* albumin, *LDH* lactate dehydrogenase

There were differences between the two groups in OS rates before the last therapy. The high-risk group showed a higher CD3 + CD8+ percentage or a lower CD3-CD56+ percentage had better OS rates. The median survival times for the high CD3 + CD8+ percentage group (average 38.50 months) and the normal CD3 + CD8+ percentage group (average 39.53 months) were significantly longer than that for the low CD3 + CD8+ percentage group (average 25.00 months) (*p* = 0.003). The median survival times for the high CD3-CD56+ percentage group (average 37.60 months) and normal CD3-CD56+ percentage group (average 40.14 months) were significantly longer than those for the low CD3-CD56+ percentage group (average 32.40 months) (*p* = 0.003). Kaplan-Meier curves of OS for lymphocyte subsets at T3 were plotted and shown in Fig. 1K-L. The prognostic values for other factors were not significant.

## Discussion

With the improved understanding of the pathogenesis of EBV associated NPC and plasma EBV DNA test for population screening as well as personalized therapy strategies, the mortality rate of NPC has been greatly reduced. However, the early diagnosis of NPC remains a challenge. Therefore, exploring therapeutic and prognostic biomarkers for NPC is necessary. In this study, NPC patients were divided into a high-risk group and a low-risk group according to the EBV DNA load before the first CRT. By comparing clinicopathological data and peripheral blood indexes between the high-risk group and the low-risk group, we hope to find potential biomarkers that can help to predict prognosis and guide NPC therapy.

In our study, the initial EBV DNA load was related to N classification that is patients with a higher initial EBV load had a higher N classification. This finding was consistent to previous studies [4, 18]. In addition, we found that EBV DNA load was associated with a variety of lymphocyte subsets, especially CD3+ percentage and CD3 + CD8+ percentage. Similar to our results, Mo et al [19] reported the relationship between EBV DNA load and CD4 + CD25+ T cells and CD8+ cells in patients with NPC. A higher CD8+ percentage tends to have a better prognosis, which may due to the fact that CD8+ cells play an important role in establishing effective immune surveillance.

Previous studies have shown that EBV DNA and LDH are independent prognostic factors for locoregionally advanced NPC [16, 17]. The circulating levels of EBV DNA and LDH in combination with other risk factors can predict recurrence and overall survival for NPC patients in established statistic nomogram models [13, 20]. In our analysis, patients with higher initial EBV load referring to high-risk group had a higher LDH level compared to the low-risk group. However, multivariate survival analyses of overall survival combining circulating lymphocyte subsets did not show the significant prognostic value of LDH in high-risk patients. Thus, additional studies are warranted to explore the role of circulating level of LDH when NPC patients are stratified by plasma EBV DNA load, ideally from large prospective studies.

During CRT, we also found that lymphocyte subset counts were decreased before last therapy compared to initial time point before therapy in both high-risk group and low-risk group. This phenomenon has been observed during CRT in other tumors. For example, Lee et al reported pancytopenia after CRT in rectal cancer [21]. It may relate to myelosuppression [22]. Schuler et al [23] reported that the number of CD4+ T cells declined after CRT in patients with head and neck cancer, because CD4+ T cells were sensitive to CRT.

Inflammation plays a crucial role in cancer development [24]. Peripheral blood neutrophils and lymphocytes not only constitute the predominant proportion, but also play an important role in the immune response. The blood neutrophil-to-lymphocyte ratio has been found to be associated with disease progression in a number of malignancies [25]. During CRT, we found NLR, PLR and SII were increased, mainly because lymphocytes were more sensitive to CRT and the decrease of lymphocytes was greater than that in other white blood cells [24].

Cancer metastases are the main cause of cancer mortality. One important question regarding which patients are likely to develop distant metastases remains to be answered. We found differences of lymphocyte subsets before therapy between the high-risk group and the low-risk group when comparing patients who developed metastases or not. For example, patients who developed metastases in the high-risk group had lower monocyte counts while CD3+ percentage, CD3-CD56+ count and CD8 + CD38+ count were decreased in the low-risk group. These differences changed after therapy. EBV may attack immune cells and interfere with the immune system cell function [26]. EBV induces the apoptosis of monocyte precursors and therefore inhibit the development of dendritic cells [27]. Our findings warrant further investigation.

CD8+ lymphocytes have been shown to play an important role in the host’s defense against malignancies, and a high infiltration of CD8+ lymphocytes was associated with good clinical outcome in many cancers [28–30]. Consistent to these findings, in the high-risk group, patients with a higher CD3 + CD8+ percentage had better OS rates and the CD3 + CD8+ percentage was an independent prognostic factor. Moreover, after treatment, patients in the high-risk group at T3 with a higher CD3-CD56+ percentage referring to the natural killer cell percentage, had poor survival compared to the low and normal CD3-CD56+ percentages. These findings suggest that the change of circulating lymphocyte subsets during treatment has the potential to predict the patient’s outcome.

In conclusion, we demonstrated that the CRT treatment caused overall decreases of blood cells in NPC patients, especially lymphocytes. We also demonstrated that patients with EBV DNA ≥ 1500 copies/mL had a higher CD3 + CD8+ percentage or a lower CD3-CD56+ percentage had better OS rates and the CD3 + CD8+ percentage was an independent prognostic factor by multivariate survival analyses. With the introduction of tumor immunotherapy as a promising therapeutic approach in NPC, our findings of the changes of circulating lymphocyte subsets during treatment may add to the current understanding of EBV-associated NPC immunity and advance the management of NPC patients.

## Supplementary Information

**Additional file 1.**

**Additional file 2.**

**Additional file 3.**

**Additional file 4.**

**Additional file 5.**

**Additional file 6.**

**Additional file 7.**

**Additional file 8.**

**Additional file 9.**

## Data Availability

The datasets generated and/or analyzed during the present study are available from the corresponding author upon reasonable request.
