# Supplementary material for: Circulating lymphocyte subsets are prognostic factors in patients with nasopharyngeal carcinoma
Source: BMC Cancer. 2022 Jun 29;22:716. doi: 10.1186/s12885-022-09438-y (PMC9241295; doi:10.1186/s12885-022-09438-y)
Supplement: Supplementary file 1 — Additional file 1. [file 12885_2022_9438_MOESM1_ESM.pdf]

Supplementary Table 1 Reagent or resource

Reagent or resource	Source	Clone	Identifier
Antibodies			
CD45-FITC/CD4-RD1/CD8-ECD/CD3-PC5	Beckman Coulter	B3821F4A/UCHT1/SFCL12T4D11/SFCL21Thy2D3	Cat#6607013
CD45-FITC/CD56-RD1/CD19-ECD/CD3-PC5	Beckman Coulter	B3821F4A/N901-NKH-1/J3-119/UCHT1	Cat#6607073
CD4-PC5	Beckman Coulter	13B8.2	Cat#A07752
CD45RA-FITC	Beckman Coulter	ALB11	Cat#A07786
CD45RO-PE	Beckman Coulter	UCHL1	Cat#A07787
CD8-PE	Beckman Coulter	B9.11	Cat#A07757
CD38-FITC	Beckman Coulter	T16	Cat#A07778
Reagent			
OptiLyse C lysing solution	Beckman Coulter	-	Cat#A11895
Coulter LH Series Diluent (PBS)	Beckman Coulter	-	Cat#8547194
Biocoll Separating Solution	Merck	-	Cat#L6155
Bovine serum albumin (BSA)	Thermo Fisher	-	Cat#23208
