# Supplementary material for: Circulating lymphocyte subsets are prognostic factors in patients with nasopharyngeal carcinoma
Source: BMC Cancer. 2022 Jun 29;22:716. doi: 10.1186/s12885-022-09438-y (PMC9241295; doi:10.1186/s12885-022-09438-y)
Supplement: Supplementary file 2 — Additional file 2. [file 12885_2022_9438_MOESM2_ESM.pdf]

Supplementary Table 2 Reference range of lymphocyte subsets.

**Lymphocytes**	**Reference range**
T cell (CD3+) percentage (%)	60.00-84.00
T cell (CD3+) count (×10^3 cells/ul)	0.97-1.80
Helper/effector T cell (CD3+CD4+) percentage (%)	29.00-60.00
Helper/effector T cell (CD3+CD4+) count (×10^3 cells/ul)	0.49-0.90
Suppressor/cytotoxic T cell (CD3+CD8+) percentage (%)	12.00-38.00
Suppressor/cytotoxic T cell (CD3+CD8+) count(×10^3 cells/ul)	0.35-0.83
CD4/CD8 ratio (CD3+CD4+/CD3+CD8+)	1.00-3.50
Natural killer cell (CD3-CD56+) percentage (%)	6.00-30.00
Natural killer cell (CD3-CD56+) count(×10^3 cells/ul)	0.18-0.62
B cell (CD3-CD19+) percentage (%)	7.00-22.00
B cell (CD3-CD19+) count(×10^3 cells/ul)	0.12-0.32
NKT cell (CD3+CD56+) percentage (%)	0.50-5.00
NKT cell (CD3+CD56+) count(×10^3 cells/ul)	0.04-0.07
*Naïve helper T cell (CD4+CD45RA+) percentage (%)	15.50-25.00
Naïve helper T cell (CD4+CD45RA+) count (×10^3 cells/ul)	0.13-0.40
*Memory helper T cell (CD4+CD45RA-) percentage (%)	9.80-26.00
Memory helper T cell (CD4+CD45RA-) count (×10^3 cells/ul)	0.27-0.59
CD4+ naive/memory ratio (CD4+CD45RA+/CD4+CD45RA-)	0.31-0.91
*Memory helper T cell (CD4+CD45RO+) percentage (%)	9.80-26.00
Memory helper T cell (CD4+CD45RO+) count (×10^3 cells/ul)	0.27-0.59
Activated CD8 cell (CD8+CD38+) percentage (%)	2.30-15.00
Activated CD8 cell (CD8+CD38+) count (×10^3 cells/ul)	0.07-0.30
Lymphocyte count (×10^3 cells/ul)	1.10-3.20
WBC count (×10^3 cells/ul)	3.50-9.50
Neutrophil count (×10^3 cells/ul)	1.80-6.30
Monocyte count (×10^3 cells/ul)	0.10-0.60
Platelet count (×10^3 cells/ul)	125.00-350.00
NLR	0.90-2.94
LMR	2.50 - 7.50
PLR	61.00 - 179.00
SII	161.00-701.00
ALB (g/L)	40.00-55.00
LDH (U/L)	0.00-240.00

*Naïve and memory T cells can be identified by the surface markers CD45RA+ and CD45RO+, respectively. After in vitro or in vivo activation, CD45RA+ cells rapidly lose CD45RA and become positive for CD45RO.

Abbreviations: NLR, neutrophil count/Lymphocyte count; LMR, lymphocyte count/monocyte count; PLR, Platelet count/Lymphocyte count; SII, Platelet count×Neutrophil count/Lymphocyte count; ALB, albumin; LDH, lactate dehydrogenase.
