# Supplementary material for: Circulating lymphocyte subsets are prognostic factors in patients with nasopharyngeal carcinoma
Source: BMC Cancer. 2022 Jun 29;22:716. doi: 10.1186/s12885-022-09438-y (PMC9241295; doi:10.1186/s12885-022-09438-y)
Supplement: Supplementary file 3 — Additional file 3. [file 12885_2022_9438_MOESM3_ESM.pdf]

Supplementary Table 3 The comparison of high-risk group and low-risk group with blood indexes over time (n=220).

	[a] **T1**			[b] **T2**			[c] **T3**		
	**High-risk group**	**Low-risk group**	***p***	**High-risk group**	**Low-risk group**	***p***	**High-risk group**	**Low-risk group**	***p***
Lymphocyte count	1.58±0.58	1.68±0.54	0.224	1.30±0.56	1.35±0.60	0.536	1.04±0.58	1.07±0.61	0.876
CD3+ %	68.50±11.62	69.41±10.39	0.558	70.67±12.45	74.07±9.95	**0.031**	69.78±13.77	71.88±10.89	0.217
CD3+ count	1.08±0.44	1.17±0.41	0.166	0.92±0.41	1.00±0.44	0.250	0.73±0.44	0.74±0.45	0.872
CD3+CD4+ %	38.27±9.25	38.26±8.50	0.994	37.85±10.59	39.60±10.09	0.239	35.91±11.52	34.42±9.83	0.318
CD3+CD4+ count	0.60±0.25	0.64±0.25	0.211	0.50±0.25	0.54±0.27	0.281	0.38±0.26	0.38±0.28	0.794
CD3+CD8+ %	24.60±8.92	26.24±8.00	0.171	27.23±9.25	29.43±9.70	0.114	27.88±10.53	31.69±9.90	**0.009**
CD3+CD8+ count	0.40±0.24	0.44±0.20	0.178	0.35±0.20	0.39±0.21	0.214	0.29±0.20	0.31±0.18	0.432
CD4/CD8 ratio	1.80±0.89	1.63±0.73	0.141	1.59±0.77	1.55±0.80	0.761	1.47±0.72	1.26±0.69	**0.037**
CD3-CD56+ %	19.02±10.39	17.92±9.41	0.434	18.30±11.25	16.59±8.96	0.226	20.07±13.30	18.51±9.97	0.342
CD3-CD56+ count	0.31±0.26	0.30±0.20	0.835	0.24±0.20	0.23±0.19	0.661	0.20±0.18	0.18±0.16	0.336
CD3-CD19+ %	9.72±5.31	9.61±4.23	0.869	7.60±6.08	6.57±3.89	0.130	7.13±6.52	6.20±4.50	0.181
CD3-CD19+ count	0.15±0.09	0.16±0.10	0.336	0.09±0.08	0.09±0.07	0.833	0.07±0.06	0.07±0.06	0.663
CD3+CD56+ %	2.98±1.87	2.78±1.95	0.943	3.25±2.11	2.90±1.70	0.188	3.40±2.12	3.10±1.96	0.290
CD3+CD56+ count	0.05±0.05	0.05±0.04	0.879	0.04±0.04	0.04±0.03	0.549	0.03±0.04	0.03±0.03	0.415
CD4+CD45RA+ %	10.04±5.11	11.24±5.84	0.140	9.28±5.28	10.72±6.96	0.124	7.25±5.85	7.46±6.52	0.813
CD4+CD45RA+ count	0.16±0.12	0.19±0.12	0.138	0.13±0.10	0.15±0.12	0.113	0.09±0.10	0.09±0.11	0.994
CD4+CD45RA- %	24.08±7.19	21.94±5.47	**0.015**	24.51±7.38	23.98±6.60	0.596	24.39±8.51	22.76±6.34	0.118
CD4+CD45RA- count	0.37±0.15	0.37±0.16	0.886	0.32±0.16	0.32±0.17	0.892	0.25±0.15	0.24±0.17	0.667
CD4+CD45RA+/CD4+CD45RA- ratio	0.45±0.25	0.54±0.34	**0.034**	0.39±0.24	0.47±0.33	0.077	0.31±0.26	0.34±0.33	0.504
CD4+CD45RO+ %	23.92±7.17	21.81±5.67	**0.019**	24.38±7.62	23.87±6.59	0.613	24.19±8.37	22.69±6.32	0.152
CD4+CD45RO+ count	0.37±0.15	0.37±0.16	0.924	0.32±0.16	0.32±0.17	0.891	0.25±0.15	0.24±0.17	0.676
CD8+CD38+ %	6.13±3.04	6.32±3.71	0.704	7.25±3.51	7.10±3.18	0.762	7.87±5.22	7.87±3.23	0.970
CD8+CD38+ count	0.10±0.07	0.11±0.07	0.498	0.09±0.06	0.10±0.07	0.606	0.08±0.07	0.08±0.05	0.771

WBC count	6.67±2.01	6.58±1.93	0.627	6.25±3.78	5.78±3.32	0.305	4.36±1.77	5.13±2.14	0.682
Neutrophil count	4.55±1.93	4.30±1.58	0.301	4.36±3.53	3.96±3.07	0.356	3.02±1.63	3.54±1.77	0.727
NLR	3.45±2.57	2.75±1.17	**0.009**	4.62±6.01	3.45±2.74	0.074	4.49±3.62	4.96±5.73	0.196
Monocyte count	0.56±0.22	0.60±0.31	0.515	0.58±0.48	0.54±0.28	0.306	0.45±0.22	0.51±0.28	0.261
LMR	3.03±1.46	3.11±1.47	0.841	2.65±2.07	2.87±2.43	0.432	2.48±1.90	2.39±1.73	0.570
Platelet count	237.80±72.25	232.54±67.16	0.283	227.96±119.66	216.37±86.63	0.262	203.20±106.69	210.57±106.98	0.123
PLR	171.99±85.28	150.42±60.16	**0.021**	212.14±137.64	201.04±144.06	0.576	294.40±236.72	287.62±316.53	0.971
SII	840.17±730.84	640.91±321.30	**0.004**	867.78±695.84	740.34±661.93	0.201	899.32±936.97	973.02±1102.79	0.723
ALB	42.46±3.90	43.74±5.37	0.066	42.19±3.30	42.51±4.78	0.570	44.50±27.07	42.63±9.31	0.383
LDH	232.69±88.74	200.84±49.60	**< 0.001**	209.43±47.12	206.92±47.40	0.252	199.25±56.30	226.03±234.44	0.070

[a] T1: before therapy. [b] T2: during therapy. [c] T3: before the last therapy. [d] Yes: patients with distant metastases. [e] No: patients without distant metastases.

Abbreviations: NLR, Neutrophil count/Lymphocyte count; LMR, Lymphocyte count/Monocyte count; PLR, Platelet count/Lymphocyte count; SII, Platelet count × Neutrophil count/Lymphocyte count; ALB, albumin; LDH, lactate dehydrogenase.
