# Supplementary material for: Circulating lymphocyte subsets are prognostic factors in patients with nasopharyngeal carcinoma
Source: BMC Cancer. 2022 Jun 29;22:716. doi: 10.1186/s12885-022-09438-y (PMC9241295; doi:10.1186/s12885-022-09438-y)
Supplement: Supplementary file 4 — Additional file 4. [file 12885_2022_9438_MOESM4_ESM.pdf]

Supplementary Table 4 Comparison between T1 and T2 in high-risk group and in low-risk group.

	**High-risk group (n=71)**			**Low-risk group (n=149)**		
	[a] **T1**	[b] **T2**	***p***	**T1**	**T2**	***p***
Lymphocyte count	1.58±0.58	1.30±0.56	**0.003**	1.68±0.54	1.35±0.60	**< 0.001**
CD3+ %	68.50±11.62	70.67±12.45	0.285	69.41±10.39	74.07±9.95	**< 0.001**
CD3+ count	1.08±0.44	0.92±0.41	**0.028**	1.17±0.41	1.00±0.44	**< 0.001**
CD3+CD4+ %	38.27±9.25	37.85±10.59	0.803	38.26±8.50	39.60±10.09	0.217
CD3+CD4+ count	0.60±0.25	0.50±0.25	**0.019**	0.64±0.25	0.54±0.27	**< 0.001**
CD3+CD8+ %	24.60±8.92	27.23±9.25	0.086	26.24±8.00	29.43±9.70	**0.002**
CD3+CD8+ count	0.40±0.24	0.35±0.20	0.209	0.44±0.20	0.39±0.21	**0.034**
CD4/CD8 ratio	1.80±0.89	1.59±0.77	0.130	1.63±0.73	1.55±0.80	0.368
CD3-CD56+ %	19.02±10.39	18.30±11.25	0.690	17.92±9.41	16.59±8.96	0.211
CD3-CD56+ count	0.31±0.26	0.24±0.20	0.070	0.30±0.20	0.23±0.19	**< 0.001**
CD3-CD19+ %	9.72±5.31	7.60±6.08	**0.028**	9.61±4.23	6.57±3.89	**< 0.001**
CD3-CD19+ count	0.15±0.09	0.09±0.08	**< 0.001**	0.16±0.10	0.09±0.07	**< 0.001**
CD3+CD56+ %	2.98±1.87	3.25±2.11	0.177	2.78±1.95	2.90±1.70	0.562
CD3+CD56+ count	0.05±0.05	0.04±0.04	0.634	0.05±0.04	0.04±0.03	0.068
CD4+CD45RA+ %	10.04±5.11	9.28±5.28	0.384	11.24±5.84	10.72±6.96	0.487
CD4+CD45RA+ count	0.16±0.12	0.13±0.10	0.056	0.19±0.12	0.15±0.12	**0.011**
CD4+CD45RA- %	24.08±7.19	24.51±7.38	0.728	21.94±5.47	23.98±6.60	**0.004**
CD4+CD45RA- count	0.37±0.15	0.32±0.16	**0.038**	0.37±0.16	0.32±0.17	**0.011**
CD4+CD45RA+/CD4+CD45RA- ratio	0.45±0.25	0.39±0.24	0.196	0.54±0.34	0.47±0.33	0.060
CD4+CD45RO+ %	23.92±7.17	24.38±7.62	0.717	21.81±5.67	23.87±6.59	**0.004**
CD4+CD45RO+ count	0.37±0.15	0.32±0.16	**0.041**	0.37±0.16	0.32±0.17	**0.012**
CD8+CD38+ %	6.13±3.04	7.25±3.51	**0.043**	6.32±3.71	7.10±3.18	0.050
CD8+CD38+ count	0.10±0.07	0.09±0.06	0.489	0.11±0.07	0.10±0.07	0.208

WBC count	6.67±2.01	6.25±3.78	0.405	6.58±1.93	5.78±3.32	**0.012**
Neutrophil count	4.55±1.93	4.36±3.53	0.700	4.30±1.58	3.96±3.07	0.223
NLR	3.45±2.57	4.62±6.01	0.132	2.75±1.17	3.45±2.74	**0.004**
Monocyte count	0.56±0.22	0.58±0.48	0.664	0.60±0.31	0.54±0.28	0.094
LMR	3.03±1.46	2.65±2.07	0.208	3.11±1.47	2.87±2.43	0.307
Platelet count	237.80±72.25	227.96±119.66	0.554	232.54±67.16	216.37±86.63	0.073
PLR	171.99±85.28	212.14±137.64	**0.039**	150.42±60.16	201.04±144.06	**< 0.001**
SII	840.17±730.84	867.78±695.84	0.818	640.91±321.30	740.34±661.93	0.100
ALB	42.46±3.90	42.19±3.30	0.662	43.74±5.37	42.51±4.78	**0.037**
LDH	232.69±88.74	209.43±47.12	0.053	200.84±49.60	206.92±47.40	0.281

[a] T1: before therapy. [b] T2: during therapy.

Abbreviations: NLR, Neutrophil count/Lymphocyte count; LMR, Lymphocyte count/Monocyte count; PLR, Platelet count/Lymphocyte count; SII, Platelet count × Neutrophil count/Lymphocyte count; ALB, albumin; LDH, lactate dehydrogenase.
