# Supplementary material for: Circulating lymphocyte subsets are prognostic factors in patients with nasopharyngeal carcinoma
Source: BMC Cancer. 2022 Jun 29;22:716. doi: 10.1186/s12885-022-09438-y (PMC9241295; doi:10.1186/s12885-022-09438-y)
Supplement: Supplementary file 5 — Additional file 5. [file 12885_2022_9438_MOESM5_ESM.pdf]

Supplementary Table 5 Comparison between T1 and T3 in high-risk group and in low-risk group.

	**High-risk group (n=71)**			**Low-risk group (n=149)**		
	[a] **T1**	[b] **T3**	***p***	**T1**	**T3**	***p***
Lymphocyte count	1.58±0.58	1.04±0.58	**< 0.001**	1.68±0.54	1.07±0.61	**< 0.001**
CD3+ %	68.50±11.62	69.78±13.77	0.549	69.41±10.39	71.88±10.89	**0.046**
CD3+ count	1.08±0.44	0.73±0.44	**< 0.001**	1.17±0.41	0.74±0.45	**< 0.001**
CD3+CD4+ %	38.27±9.25	35.91±11.52	0.181	38.26±8.50	34.42±9.83	**< 0.001**
CD3+CD4+ count	0.60±0.25	0.38±0.26	**< 0.001**	0.64±0.25	0.38±0.28	**< 0.001**
CD3+CD8+ %	24.60±8.92	27.88±10.53	**0.047**	26.24±8.00	31.69±9.90	**< 0.001**
CD3+CD8+ count	0.40±0.24	0.29±0.20	**0.004**	0.44±0.20	0.31±0.18	**< 0.001**
CD4/CD8 ratio	1.80±0.89	1.47±0.72	**0.016**	1.63±0.73	1.26±0.69	**< 0.001**
CD3-CD56+ %	19.02±10.39	20.07±13.30	0.601	17.92±9.41	18.51±9.97	0.599
CD3-CD56+ count	0.31±0.26	0.20±0.18	**0.005**	0.30±0.20	0.18±0.16	**< 0.001**
CD3-CD19+ %	9.72±5.31	7.13±6.52	**0.011**	9.61±4.23	6.20±4.50	**< 0.001**
CD3-CD19+ count	0.15±0.09	0.07±0.06	**< 0.001**	0.16±0.10	0.07±0.06	**< 0.001**
CD3+CD56+ %	2.98±1.87	3.40±2.12	0.074	2.78±1.95	3.10±1.96	0.160
CD3+CD56+ count	0.05±0.05	0.03±0.04	0.122	0.05±0.04	0.03±0.03	**< 0.001**
CD4+CD45RA+ %	10.04±5.11	7.25±5.85	**0.003**	11.24±5.84	7.46±6.52	**< 0.001**
CD4+CD45RA+ count	0.16±0.12	0.09±0.10	**< 0.001**	0.19±0.12	0.09±0.11	**< 0.001**
CD4+CD45RA- %	24.08±7.19	24.39±8.51	0.820	21.94±5.47	22.76±6.34	0.236
CD4+CD45RA- count	0.37±0.15	0.25±0.15	**< 0.001**	0.37±0.16	0.24±0.17	**< 0.001**
CD4+CD45RA+/CD4+CD45RA- ratio	0.45±0.25	0.31±0.26	**0.002**	0.54±0.34	0.34±0.33	**< 0.001**
CD4+CD45RO+ %	23.92±7.17	24.19±8.37	0.841	21.81±5.67	22.69±6.32	0.233
CD4+CD45RO+ count	0.37±0.15	0.25±0.15	**< 0.001**	0.37±0.16	0.24±0.17	**< 0.001**
CD8+CD38+ %	6.13±3.04	7.87±5.22	**0.016**	6.32±3.71	7.87±3.23	**< 0.001**
CD8+CD38+ count	0.10±0.07	0.08±0.07	0.114	0.11±0.07	0.08±0.05	**< 0.001**

WBC count	6.67±2.01	4.36±1.77	**< 0.001**	6.58±1.93	5.13±2.14	**< 0.001**
Neutrophil count	4.55±1.93	3.02±1.63	**< 0.001**	4.30±1.58	3.54±1.77	**< 0.001**
NLR	3.45±2.57	4.49±3.62	0.051	2.75±1.17	4.96±5.73	**< 0.001**
Monocyte count	0.56±0.22	0.45±0.22	**0.004**	0.60±0.31	0.51±0.28	**0.015**
LMR	3.03±1.46	2.48±1.90	0.054	3.11±1.47	2.39±1.73	**< 0.001**
Platelet count	237.80±72.25	203.20±106.69	**0.025**	232.54±67.16	210.57±106.98	**0.035**
PLR	171.99±85.28	294.40±236.72	**< 0.001**	150.42±60.16	287.62±316.53	**< 0.001**
SII	840.17±730.84	899.32±936.97	0.676	640.91±321.30	973.02±1102.79	**< 0.001**
ALB	42.46±3.90	44.50±27.07	0.531	43.74±5.37	42.63±9.31	0.205
LDH	232.69±88.74	199.25±56.30	**0.008**	200.84±49.60	226.03±234.44	0.200

[a] T1: before therapy. [b] T3: before the last therapy.

Abbreviations: NLR, Neutrophil count/Lymphocyte count; LMR, Lymphocyte count/Monocyte count; PLR, Platelet count/Lymphocyte count; SII, Platelet count × Neutrophil count/Lymphocyte count; ALB, albumin; LDH, lactate dehydrogenase.
