# Supplementary material for: Circulating lymphocyte subsets are prognostic factors in patients with nasopharyngeal carcinoma
Source: BMC Cancer. 2022 Jun 29;22:716. doi: 10.1186/s12885-022-09438-y (PMC9241295; doi:10.1186/s12885-022-09438-y)
Supplement: Supplementary file 6 — Additional file 6. [file 12885_2022_9438_MOESM6_ESM.pdf]

Supplementary Table 6 Comparison between T2 and T3 in high-risk group and in low-risk group.

	**High-risk group (n=71)**			**Low-risk group (n=149)**		
	[a] **T2**	[b] **T3**	***p***	**T2**	**T3**	***p***
Lymphocyte count	1.30±0.56	1.04±0.58	**0.007**	1.35±0.60	1.07±0.61	**< 0.001**
CD3+ %	70.67±12.45	69.78±13.77	0.688	74.07±9.95	71.88±10.89	0.072
CD3+ count	0.92±0.41	0.73±0.44	**0.008**	1.00±0.44	0.74±0.45	**< 0.001**
CD3+CD4+ %	37.85±10.59	35.91±11.52	0.298	39.60±10.09	34.42±9.83	**< 0.001**
CD3+CD4+ count	0.50±0.25	0.38±0.26	**0.008**	0.54±0.27	0.38±0.28	**< 0.001**
CD3+CD8+ %	27.23±9.25	27.88±10.53	0.699	29.43±9.70	31.69±9.90	**0.047**
CD3+CD8+ count	0.35±0.20	0.29±0.20	0.065	0.39±0.21	0.31±0.18	**< 0.001**
CD4/CD8 ratio	1.59±0.77	1.47±0.72	0.341	1.55±0.80	1.26±0.69	**< 0.001**
CD3-CD56+ %	18.30±11.25	20.07±13.30	0.391	16.59±8.96	18.51±9.97	0.081
CD3-CD56+ count	0.24±0.20	0.20±0.18	0.275	0.23±0.19	0.18±0.16	**0.030**
CD3-CD19+ %	7.60±6.08	7.13±6.52	0.662	6.57±3.89	6.20±4.50	0.450
CD3-CD19+ count	0.09±0.08	0.07±0.06	**0.045**	0.09±0.07	0.07±0.06	**< 0.001**
CD3+CD56+ %	3.25±2.11	3.40±2.12	0.671	2.90±1.70	3.10±1.96	0.357
CD3+CD56+ count	0.04±0.04	0.03±0.04	0.223	0.04±0.03	0.03±0.03	**0.013**
CD4+CD45RA+ %	9.28±5.28	7.25±5.85	**0.032**	10.72±6.96	7.46±6.52	**< 0.001**
CD4+CD45RA+ count	0.13±0.10	0.09±0.10	**0.030**	0.15±0.12	0.09±0.11	**< 0.001**
CD4+CD45RA- %	24.51±7.38	24.39±8.51	0.925	23.98±6.60	22.76±6.34	0.102
CD4+CD45RA- count	0.32±0.16	0.25±0.15	**0.010**	0.32±0.17	0.24±0.17	**< 0.001**
CD4+CD45RA+/CD4+CD45RA- ratio	0.39±0.24	0.31±0.26	0.051	0.47±0.33	0.34±0.33	**< 0.001**
CD4+CD45RO+ %	24.38±7.62	24.19±8.37	0.889	23.87±6.59	22.69±6.32	0.101
CD4+CD45RO+ count	0.32±0.16	0.25±0.15	**0.011**	0.32±0.17	0.24±0.17	**< 0.001**
CD8+CD38+ %	7.25±3.51	7.87±5.22	0.409	7.10±3.18	7.87±3.23	**0.039**
CD8+CD38+ count	0.09±0.06	0.08±0.07	0.294	0.10±0.07	0.08±0.05	**0.008**

WBC count	6.25±3.78	4.36±1.77	**< 0.001**	5.78±3.32	5.13±2.14	**0.045**
Neutrophil count	4.36±3.53	3.02±1.63	**0.004**	3.96±3.07	3.54±1.77	0.153
NLR	4.62±6.01	4.49±3.62	0.869	3.45±2.74	4.96±5.73	**0.004**
Monocyte count	0.58±0.48	0.45±0.22	**0.031**	0.54±0.28	0.51±0.28	0.428
LMR	2.65±2.07	2.48±1.90	0.606	2.87±2.43	2.39±1.73	**0.048**
Platelet count	227.96±119.66	203.20±106.69	0.195	216.37±86.63	210.57±106.98	0.608
PLR	212.14±137.64	294.40±236.72	**0.013**	201.04±144.06	287.62±316.53	**0.003**
SII	867.78±695.84	899.32±936.97	0.820	740.34±661.93	973.02±1102.79	**0.028**
ALB	42.19±3.30	44.50±27.07	0.478	42.51±4.78	42.63±9.31	0.894
LDH	209.43±47.12	199.25±56.30	0.245	206.92±47.40	226.03±234.44	0.330

[a] T1: before therapy. [b] T3: before the last therapy.

Abbreviations: NLR, Neutrophil count/Lymphocyte count; LMR, Lymphocyte count/Monocyte count; PLR, Platelet count/Lymphocyte count; SII, Platelet count × Neutrophil count/Lymphocyte count; ALB, albumin; LDH, lactate dehydrogenase.
