# Supplementary material for: Circulating lymphocyte subsets are prognostic factors in patients with nasopharyngeal carcinoma
Source: BMC Cancer. 2022 Jun 29;22:716. doi: 10.1186/s12885-022-09438-y (PMC9241295; doi:10.1186/s12885-022-09438-y)
Supplement: Supplementary file 7 — Additional file 7. [file 12885_2022_9438_MOESM7_ESM.pdf]

Supplementary Table 7 Comparison of blood indexes in patients with or without metastases in high-risk group at different time points (n=71).

**Parameters**	[a] **T1**			[b] **T2**			[c] **T3**		
	[d] **Yes**	[e] **No**	***p***	**Yes**	**No**	***p***	**Yes**	**No**	***p***
Lymphocyte count	1.41±0.55	1.63±0.58	0.204	1.33±0.72	1.33±0.57	0.979	0.97±0.58	1.05±0.58	0.668
CD3+ %	65.38±13.52	69.27±11.11	0.265	67.89±16.66	71.35±11.27	0.354	67.59±12.74	70.32±14.06	0.510
CD3+ count	0.91±0.39	1.12±0.44	0.108	0.82±0.45	0.95±0.40	0.304	0.66±0.38	0.75±0.46	0.473
CD3+CD4+ %	37.08±12.32	38.56±8.45	0.595	37.03±14.14	38.05±9.68	0.748	34.75±6.19	36.20±12.51	0.676
CD3+CD4+ count	0.53±0.29	0.62±0.24	0.247	0.46±0.27	0.51±0.25	0.490	0.35±0.22	0.39±0.27	0.555
CD3+CD8+ %	22.75±5.60	25.05±9.55	0.391	27.02±11.37	27.28±8.76	0.925	27.47±9..95	27.98±10.75	0.873
CD3+CD8+ count	0.32±0.15	0.42±0.25	0.151	0.31±0.19	0.36±0.20	0.339	0.26±0.16	0.30±0.20	0.477
CD4/CD8 ratio	1.79±0.92	1.81±0.89	0.942	1.63±1.00	1.58±0.71	0.831	1.40±0.50	1.49±0.77	0.693
CD3-CD56+ %	22.23±12.17	18.24±9.87	0.200	20.65±14.66	17.72±10.32	0.386	23.16±10.68	19.31±13.84	0.335
CD3-CD56+ count	0.32±0.28	0.31±0.26	0.878	0.26±0.23	0.24±0.19	0.703	0.25±0.19	0.19±0.18	0.326
CD3-CD19+ %	8.94±6.67	9.91±4.97	0.546	7.46±7.46	7.63±5.77	0.924	6.96±6.16	7.18±6.66	0.911
CD3-CD19+ count	0.13±0.14	0.15±0.08	0.497	0.10±0.16	0.09±0.05	0.670	0.07±0.09	0.07±0.05	0.823
CD3+CD56+ %	2.51±1.32	2.87±1.98	0.520	3.19±2.45	3.27±2.04	0.899	2.84±2.82	3.54±1.91	0.267
CD3+CD56+ count	0.04±0.03	0.05±0.05	0.391	0.03±0.02	0.04±0.04	0.413	0.02±0.02	0.04±0.04	0.219
CD4+CD45RA+ %	9.20±5.12	10.25±5.13	0.496	9.12±6.60	9.32±4.97	0.901	5.96±4.96	7.57±6.05	0.358
CD4+CD45RA+ count	0.13±0.10	0.17±0.12	0.304	0.12±0.11	0.13±0.09	0.808	0.07±0.08	0.10±0.10	0.428
CD4+CD45RA- %	23.82±7.49	24.15±7.18	0.880	24.11±8.18	24.61±7.24	0.821	24.24±5.65	24.42±9.12	0.945
CD4+CD45RA- count	0.35±0.19	0.38±0.14	0.432	0.29±0.16	0.33±0.16	0.512	0.24±0.15	0.25±0.16	0.888
CD4+CD45RA+/CD4+CD45RA- ratio	0.37±0.17	0.47±0.27	0.233	0.35±0.22	0.40±0.24	0.481	0.26±0.21	0.32±0.28	0.398
CD4+CD45RO+ %	23.52±7.38	24.02±7.18	0.879	24.15±8.08	24.51±7.84	0.819	24.05±5.52	24.22±8.97	0.424
CD4+CD45RO+ count	0.34±0.19	0.38±0.14	0.431	0.28±0.12	0.31±0.13	0.511	0.24±0.15	0.25±0.16	0.941
CD8+CD38+ %	5.81±2.96	6.20±3.08	0.671	7.75±4.39	7.13±3.30	0.555	7.72±2.49	7.90±5.71	0.908
CD8+CD38+ count	0.08±0.07	0.10±0.08	0.358	0.09±0.08	0.09±0.06	0.777	0.07±0.04	0.08±0.08	0.678

WBC count	6.79±2.44	6.69±1.92	0.869	7.49±5.21	6.02±3.84	0.237	5.31±1.88	4.72±1.64	0.240
Neutrophil count	4.94±2.58	4.46±1.76	0.419	5.65±4.92	4.10±3.54	0.180	3.83±1.78	3.22±1.44	0.180
NLR	4.10±2.87	3.24±2.48	0.266	5.30±4.09	4.32±6.46	0.591	6.99±6.63	5.09±6.78	0.349
Monocyte count	0.46±0.18	0.60±0.24	**0.043**	0.59±0.45	0.60±0.50	0.941	0.51±0.16	0.45±0.18	0.251
LMR	2.86±1.57	3.02±1.41	0.706	2.66±3.30	2.64±1.63	0.974	1.82±0.88	2.85±2.41	0.123
Platelet count	222.36±77.17	248.33±70.58	0.230	253.43±197.94	227.07±91.17	0.460	176.21±55.25	195.04±113.34	0.549
PLR	178.34±94.49	172.47±84.10	0.820	233.91±168.99	207.28±130.80	0.522	284.77±228.35	288.09±320.52	0.971
SII	958.44±850.59	818.11±698.73	0.521	1105.08±778.10	807.03±687.99	0.161	1030.17±859.93	975.07±1292.09	0.880
ALB	41.14±4.73	42.75±3.54	0.158	40.93±3.97	42.45±3.18	0.313	40.63±5.06	42.06±4.32	0.287
LDH	266.00±117.87	225.53±78.70	0.125	249.86±119.07	207.83±49.38	**0.043**	408.71±747.19	213.65±58.85	0.051

[a] T1: before therapy. [b] T2: during therapy. [c] T3: before the last therapy. [d] Yes: patients with distant metastases. [e] No: patients without distant metastases.

Abbreviations: NLR, Neutrophil count/Lymphocyte count; LMR, Lymphocyte count/Monocyte count; PLR, Platelet count/Lymphocyte count; SII, Platelet count × Neutrophil count/Lymphocyte count; ALB, albumin; LDH, lactate dehydrogenase.
