# Supplementary material for: Circulating lymphocyte subsets are prognostic factors in patients with nasopharyngeal carcinoma
Source: BMC Cancer. 2022 Jun 29;22:716. doi: 10.1186/s12885-022-09438-y (PMC9241295; doi:10.1186/s12885-022-09438-y)
Supplement: Supplementary file 8 — Additional file 8. [file 12885_2022_9438_MOESM8_ESM.pdf]

Supplementary Table 8 Comparison of blood indexes in patients with or without metastases in low-risk group at different time points (n=149).

**Parameters**	[a] **T1**			[b] **T2**			[c] **T3**		
	[d] **Yes**	[e] **No**	***p***	**Yes**	**No**	***p***	**Yes**	**No**	***p***
Lymphocyte count	1.52±0.50	1.73±0.54	0.076	1.21±0.53	1.37±0.61	0.109	0.95±0.61	1.06±0.58	0.347
CD3+ %	72.47±9.44	68.64±10.47	**0.035**	75.68±10.32	73.92±9.68	0.252	70.27±11.69	72.97±10.52	0.538
CD3+ count	1.11±0.41	1.18±0.41	0.429	0.92±0.48	1.02±0.43	0.293	0.68±0.51	0.76±0.44	0.378
CD3+CD4+ %	38.96±8.02	38.08±8.61	0.482	39.43±9.36	39.60±10.31	0.910	32.27±8.49	34.06±9.72	0.293
CD3+CD4+ count	0.6±0.23	0.66±0.26	0.305	0.48±0.26	0.56±0.28	0.151	0.35±0.30	0.38±0.27	0.362
CD3+CD8+ %	27.83±8.96	25.84±7.68	0.136	30.36±10.20	28.92±9.53	0.187	32.75±9.70	31.35±9.83	0.674
CD3+CD8+ count	0.43±0.23	0.44±0.2	0.926	0.38±0.23	0.39±0.20	0.932	0.30±0.20	0.32±0.18	0.542
CD4/CD8 ratio	1.64±0.84	1.63±0.7	0.865	1.49±0.80	1.58±0.80	0.528	1.10±0.65	1.32±1.48	0.138
CD3-CD56+ %	15.54±6.88	18.52±9.87	0.066	15.39±8.62	16.94±8.91	0.284	19.25±10.58	18.69±9.25	0.679
CD3-CD56+ count	0.24±0.14	0.32±0.21	**0.043**	0.17±0.16	0.24±0.20	0.078	0.16±0.14	0.19±0.16	0.418
CD3-CD19+ %	9.19±5.32	9.71±3.89	0.542	6.32±5.24	6.66±3.85	0.493	6.51±6.01	6.17±4.11	0.696
CD3-CD19+ count	0.14±0.11	0.17±0.09	0.131	0.07±0.06	0.10±0.07	**0.026**	0.06±0.08	0.07±0.06	0.826
CD3+CD56+ %	2.92±1.57	2.74±2.03	0.427	3.17±1.35	2.86±1.73	0.274	3.06±1.49	3.11±2.56	0.940
CD3+CD56+ count	0.04±0.03	0.05±0.04	0.848	0.04±0.03	0.04±0.03	0.977	0.03±0.03	0.03±0.02	0.946
CD4+CD45RA+ %	11.67±5.98	11.13±5.8	0.682	10.75±6.73	10.72±7.01	0.974	5.97±4.42	7.52±6.78	0.312
CD4+CD45RA+ count	0.17±0.1	0.19±0.12	0.389	0.14±0.09	0.16±0.11	0.321	0.07±0.14	0.10±0.13	0.170
CD4+CD45RA- %	22.11±4.35	21.9±5.71	0.610	24.04±5.35	23.92±6.46	0.925	21.53±5.96	23.42±6.38	0.289
CD4+CD45RA- count	0.34±0.15	0.38±0.16	0.395	0.29±0.16	0.33±0.17	0.194	0.22±0.19	0.24±0.16	0.396
CD4+CD45RA+/CD4+CD45RA- ratio	0.55±0.3	0.54±0.35	0.822	0.45±0.29	0.47±0.33	0.819	0.29±0.20	0.34±0.31	0.264
CD4+CD45RO+ %	22.03±4.52	21.75±5.92	0.631	24.20±5.33	23.79±6.72	0.911	21.38±5.71	22.69±6.95	0.267
CD4+CD45RO+ count	0.34±0.15	0.38±0.16	0.370	0.29±0.14	0.32±0.19	0.181	0.22±0.18	0.24±0.17	0.327
CD8+CD38+ %	5.43±2.32	6.54±3.95	0.125	6.78±3.01	7.25±3.20	0.489	7.59±2.39	7.62±3.04	0.473
CD8+CD38+ count	0.08±0.04	0.11±0.08	**0.034**	0.07±0.12	0.10±0.46	**0.042**	0.06±0.04	0.08±0.05	0.107

WBC count	6.30±1.44	6.64±1.66	0.361	5.72±3.15	5.80±3.36	0.832	5.10±2.72	4.96±2.49	0.684
Neutrophil count	4.15±1.34	4.34±1.40	0.431	3.90±2.98	3.95±3.16	0.923	3.60±1.49	3.41±1.83	0.582
NLR	2.81±0.93	2.70±1.21	0.536	3.75±2.52	3.42±2.73	0.443	5.17±5.28	4.84±3.49	0.633
Monocyte count	0.63±0.32	0.59±0.31	0.514	0.56±0.28	0.54±0.29	0.629	0.55±0.32	0.50±0.29	0.264
LMR	2.95±1.29	3.16±1.51	0.450	2.27±1.36	3.02±2.62	0.125	2.32±1.27	2.31±1.48	0.327
Platelet count	242.65±70.25	229.68±67.33	0.343	227.86±101.86	213.83±82.16	0.131	227.15±120.27	216.16±101.48	0.456
PLR	176.91±79.26	143.74±51.88	**0.007**	246.38±153.83	189.27±139.51	**0.030**	300.26±170.51	287.50±314.26	0.793
SII	693.35±268.25	627.68±331.19	0.319	905.74±795.24	699.42±583.27	0.098	1046.16±858.63	902.64±972.64	0.393
ALB	44.31±6.22	43.60±5.34	0.474	43.95±5.62	42.96±4.77	0.379	46.25±18.74	43.96±21.82	0.472
LDH	198.87±58.36	201.34±47.89	0.824	213.38±45.63	202.53±48.52	0.668	210.16±73.49	198.61±41.47	0.168

[a] T1: before therapy. [b] T2: during therapy. [c] T3: before the last therapy. [d] Yes: patients with distant metastases. [e] No: patients without distant metastases.

Abbreviations: NLR, Neutrophil count/Lymphocyte count; LMR, Lymphocyte count/Monocyte count; PLR, Platelet count/Lymphocyte count; SII, Platelet count × Neutrophil count/Lymphocyte count; ALB, albumin; LDH, lactate dehydrogenase.
