# Supplementary material for: Circulating lymphocyte subsets are prognostic factors in patients with nasopharyngeal carcinoma
Source: BMC Cancer. 2022 Jun 29;22:716. doi: 10.1186/s12885-022-09438-y (PMC9241295; doi:10.1186/s12885-022-09438-y)
Supplement: Supplementary file 9 — Additional file 9. [file 12885_2022_9438_MOESM9_ESM.pdf]

Supplementary Table 9 Univariate and multivariate survival analyses of overall survival in low-risk group (n=149).

Variable	Univariate		Multivariate	
	p	**HR (95% CI)**	***p***	**HR (95% CI)**
Age	**0.005**	1.078 (1.022-1.136)	**0.005**	1.089 (1.026-1.156)
Sex	0.553	1.346 (0.504-3.591)		
Smoking	0.488	0.719 (0.284-1.826)		
Drinking	0.772	0.858 (0.305-2.414)		
Family history of cancer	0.482	0.690 (0.246-1.937)		
pT classification	0.441	1.264 (0.696-2.297)		
pN classification	0.752	1.105(0.596-2.049)		
UICC stage	0.711	1.148 (0.554-2.378)		
Distant metastases	0.610	1.308 (0.466-3.673)		
Lymphocyte count	0.838	0.920 (0.412-2.050)		
CD3+ %	0.879	0.997 (0.957-1.038)		
CD3+ count	0.854	0.902 (0.302-2.695)		
CD3+CD4+ %	0.082	1.043 (0.995-1.094)		
CD3+CD4+ count	0.502	1.695 (0.364-7.897)		
CD3+CD8+ %	0.114	0.961 (0.914-1.010)		
CD3+CD8+ count	0.174	0.117 (0.005-2.589)		
CD4/CD8 ratio	**0.032**	1.834 (1.054-3.192)	0.788	0.903 (0.428-1.904)
CD3-CD56+ %	0.947	1.001 (0.960-1.045)		
CD3-CD56+ count	0.949	1.095 (0.068-17.670)		
CD3-CD19+ %	0.864	0.991 (0.896-1.097)		
CD3-CD19+ count	0.343	3.111 (0.350-6.696)		
CD3+CD56+ %	0.380	0.888 (0.682-1.157)		
CD3+CD56+ count	0.733	3.036 (0.488-8.676)		
CD4+CD45RA+ %	0.478	1.024 (0.959-1.094)		
CD4+CD45RA+ count	0.890	1.352 (0.019-96.634)		
CD4+CD45RA- %	**0.025**	1.083 (1.010-1.160)	0.487	0.791 (0.409-1.531)
CD4+CD45RA- count	0.414	2.826 (0.233-34.207)		
CD4+CD45RA+/CD4+CD45RA- ratio	0.880	1.115 (0.269-4.627)		
CD4+CD45RO+ %	**0.018**	1.090 (1.015-1.171)	0.306	1.414 (0.729-2.742)
CD4+CD45RO+ count	0.408	2.872 (0.236-34.944)		
CD8+CD38+ %	0.909	0.992 (0.859-1.145)		
CD8+CD38+ count	0.707	0.874 (0.730-1.937)		
WBC count	0.160	1.140 (0.949-1.370)		
Neutrophil count	0.062	1.226 (0.990-1.518)		
NLR	0.580	1.020 (0.950-1.096)		
Monocyte count	0.496	0.507 (0.072-3.579)		
LMR	0.329	1.121 (0.891-1.410)		
Platelet count	**0.042**	1.003 (1.000-1.007)	**0.030**	1.004 (1.000-1.008)
PLR	0.912	1.000 (0.999-1.001)		
SII	0.452	1.000 (1.000-1.000)		
ALB	0.625	0.981(0.907-1.060)		

LDH	0.889	1.000 (0.998-1.002)

Abbreviations: HR, hazard ratio; CI, confidence interval; NLR, Neutrophil count/Lymphocyte count; LMR, Lymphocyte count/Monocyte count; PLR, Platelet count/Lymphocyte count; SII, Platelet count × Neutrophil count/Lymphocyte count; ALB, albumin; LDH, lactate dehydrogenase.
